# Biosynthesis of the sactipeptide Ruminococcin C by the human microbiome: Mechanistic insights into thioether bond formation by radical SAM enzymes

**DOI:** 10.1074/jbc.RA120.015371

**Published:** 2021-01-13

**Authors:** Clémence Balty, Alain Guillot, Laura Fradale, Clémence Brewee, Benjamin Lefranc, Christian Herrero, Corine Sandström, Jérôme Leprince, Olivier Berteau, Alhosna Benjdia

**Affiliations:** 1 Micalis Institute, ChemSyBio, INRAE, AgroParisTech, Université Paris-Saclay, Jouy-en-Josas, France; 2 INSERM U1239, PRIMACEN, Université de Normandie-Rouen, Rouen, France; 3 ICMMO, CNRS, Université Paris-Saclay, Orsay, France; 4 Department of Molecular Sciences, Uppsala BioCenter, Swedish University of Agricultural Sciences, Uppsala, Sweden

**Keywords:** radical SAM enzyme, radical AdoMet enzyme, antimicrobial peptide, microbiota, microbiome, antibiotics, enzyme, peptide biosynthesis, RiPP, ruminococcin C, RumC, sactipeptide, antimicrobial peptide (AMP), metalloenzyme, radical, enzyme catalysis

## Abstract

Despite its major importance in human health, the metabolic potential of the human gut microbiota is still poorly understood. We have recently shown that biosynthesis of Ruminococcin C (RumC), a novel ribosomally synthesized and posttranslationally modified peptide (RiPP) produced by the commensal bacterium *Ruminococcus gnavus*, requires two radical SAM enzymes (RumMC1 and RumMC2) catalyzing the formation of four C_α_-thioether bridges. These bridges, which are essential for RumC's antibiotic properties against human pathogens such as *Clostridium perfringens*, define two hairpin domains giving this sactipeptide (sulfur-to-α-carbon thioether–containing peptide) an unusual architecture among natural products. We report here the biochemical and spectroscopic characterizations of RumMC2. EPR spectroscopy and mutagenesis data support that RumMC2 is a member of the large family of SPASM domain radical SAM enzymes characterized by the presence of three [4Fe-4S] clusters. We also demonstrate that this enzyme initiates its reaction by C_α_ H-atom abstraction and is able to catalyze the formation of nonnatural thioether bonds in engineered peptide substrates. Unexpectedly, our data support the formation of a ketoimine rather than an α,β-dehydro-amino acid intermediate during C_α_-thioether bridge LC–MS/MS fragmentation. Finally, we explored the roles of the leader peptide and of the RiPP precursor peptide recognition element, present in myriad RiPP-modifying enzymes. Collectively, our data support a more complex role for the peptide recognition element and the core peptide for the installation of posttranslational modifications in RiPPs than previously anticipated and suggest a possible reaction intermediate for thioether bond formation.

The human microbiome represents an untapped but promising source of antibiotics (1, 2, 3). Recently, several novel antibiotics such as colicin V (4), humimycin (5), and ruminococcin C (RumC) (6, 7) have been characterized from this complex environment. In a fascinating manner, these novel antibiotics belong to the emerging family of ribosomally synthesized and posttranslationally modified peptides (RiPPs) (8, 9) that encompasses a large diversity of architectures from the linear epipeptide (10) to the cyclic darobactin (11). Although these RiPPs are produced by different bacterial species, some inhabitants of the human digestive tract such as *Ruminococcus gnavus* have been shown to produce unrelated classes of RiPPs including the lanthipeptide ruminococcin A (RumA) (12) and the sactipeptide RumC (7). Intriguingly, RumA and RumC, although belonging to two distinct RiPP families, target *Clostridium perfringens* and related Gram-positive bacteria (7, 13, 14), calling to question the relevance of such apparently redundant systems.

Despite a simple biosynthetic logic based on the translation of a precursor peptide, followed by the installation of posttranslational modifications by tailoring enzymes and the cleavage of a leader and/or follower sequence during the export in the external medium, RiPPs have evolved an outstanding structural diversity (8, 15, 16). This diversity is largely due to the various and unrelated enzyme families that install posttranslational modifications in the precursor peptides. Among them, radical SAM enzymes have recently emerged as key catalysts (8, 9, 17, 18, 19, 20, 21). These enzymes are involved in the catalysis of chemically unrelated transformations such as methylation (22, 23, 24), peptide epimerization (1, 25, 26, 27), complex rearrangements (28, 29, 30), and carbon-carbon (10, 31) and thioether bridge formation (32, 33, 34, 35). Interestingly, whereas most radical SAM enzymes installing thioether bridges have been shown to catalyze the formation of sulfur-to-α carbon thioether bonds in the so-called “sactipeptides,” novel radical SAM enzymes have been recently described installing sulfur-to-β- and sulfur-to-γ-carbon thioether bridges, further expanding the catalytic diversity of radical SAM enzymes (36). To date, two mechanisms have been proposed for C_α_-thioether bridge formation. The first mechanism involves, after H-atom abstraction, formation of a ketoimine intermediate followed by a polar reaction (32), whereas the second one implies trapping of the C_α_ radical intermediate by a cysteine residue coordinated to an auxiliary iron-sulfur cluster (33). Indeed, early studies have shown that radical SAM enzymes involved in protein (37, 38) or peptide posttranslational modification often possess a so-called SPASM/twitch domain containing auxiliary iron-sulfur clusters (39, 40, 41). The function of these iron-sulfur clusters is ill-understood, but they are required for catalysis. In addition, like myriad posttranslational modification enzymes, radical SAM enzymes usually contain in their N terminus a RiPP precursor peptide recognition element (RRE) (42) whose function is presumed to be key for enzyme-peptide recognition and interaction.

To gain insights into the role of the RRE and SPASM domains and thioether bond catalysis, we undertook the biochemical characterization of RumMC2 that we have recently shown installs four C_α_-thioether bridges in RumC (7) (Fig. 1*a*).

**Figure 1:**
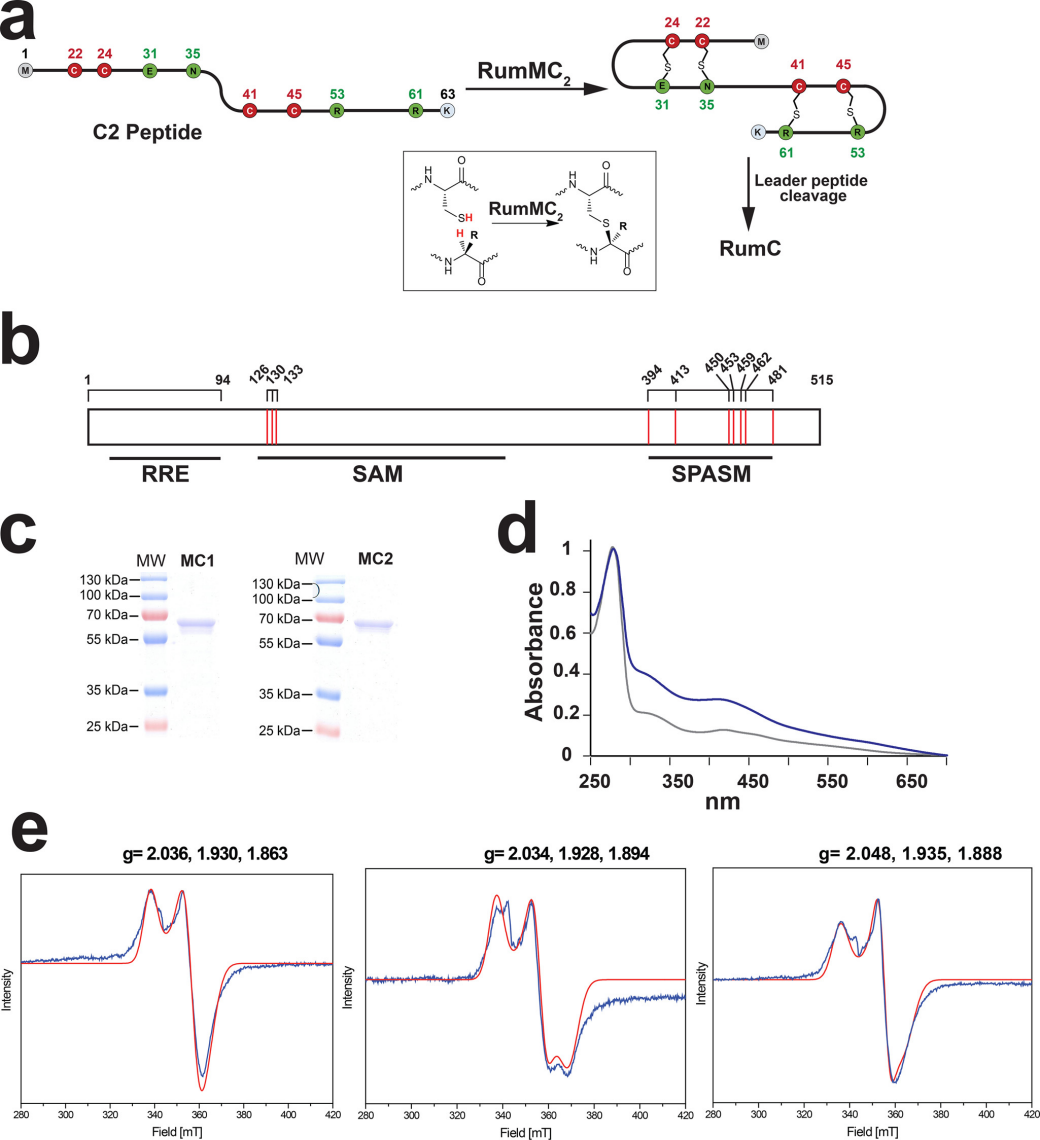
**RumMC1 and RumMC2 reaction, purification, and spectroscopic characterization.***a*, biosynthesis of RumC. After translation, the C2 peptide is modified by the radical SAM enzyme RumMC2 which installs four thioether bridges. Cleavage of the leader peptide (residues Met^1^ to Lys^19^) leads to the release of the mature RumC. In *red* and *green* are indicated cysteine residues and other amino acid residues involved in thioether bridges, respectively. Numbering refers to the position of amino acid residues in the full-length peptide. In *inset*, reaction catalyzed by RumMC2 and RumMC1. *b*, domain prediction of the radical SAM enzyme RumMC2. Based on HHpred server analysis, RumMC2 is predicted to contain an RRE (residues 16–94), a radical SAM (residues 120–297), and a SPASM domain (residues 394–481). *Red vertical bars* indicate the cysteine residues (*numbered*) predicted to be involved in the coordination of [4Fe-4S] clusters. *c*, SDS-PAGE analysis of RumMC1 (*MC1*) and RumMC2 (*MC2*). *d*, UV-visible spectra of RumMC2 before (*gray trace*) and after (*blue trace*) iron-sulfur cluster reconstitution. *e*, EPR spectra of RumMC2 alone (*left panel*), of RumMC2 in the presence of SAM (*middle panel*), and of the A3 mutant (*right panel*). All samples were reduced by sodium dithionite (3 mm). *Blue traces*: experimental data; *Red traces*: simulation. Microwave frequency = 9.636 GHz, microwave power = 1.0 milliwatt, modulation amplitude = 8 Gauss, modulation frequency = 100 KHz, Gain = 40 db, temperature =10 K.

## Results

### RumMC1 and RumMC2 are radical SAM enzymes with three [4Fe-4S] clusters

The RumC biosynthetic cluster contains two putative radical SAM enzymes (RumMC1 and RumMC2). In addition to the three cysteine residues defining the canonical CX3CX2C radical SAM motif, sequence analysis revealed the presence of seven cysteine residues clustered in a characteristic motif, C*X*13G*X*4C*X*36C*X*2C*X*5C*X*2C*X*18C, in the C-terminal end of RumMC1 and RumMC2. This motif is the hallmark of the so-called SPASM domain (subtilosin A, pyrroloquinoline quinone, anaerobic sulfatase, and mycofactocin domain) radical SAM enzymes (Fig. S1) (41). Initially identified in the anaerobic sulfatase-maturating enzyme (anSME) which catalyzes the oxidation of a critical catalytic residue in sulfatases (37, 38, 43, 44, 45, 46), this domain is widespread among radical SAM enzymes catalyzing peptide posttranslational modification such AlbA (32, 33), KW cyclase (10, 47), and PqqE (48).

In addition to the SPASM domain, sequence analysis, using the HHPred server, revealed the presence of a putative RRE domain between the residues 16–94 in both enzymes. This domain, encountered in myriad enzymes, is postulated to interact with the leader peptide for the correct installation of posttranslational modifications. Hence, RumMC1 and RumMC2 were predicted to contain three distinct domains, including an RRE, a radical SAM, and a SPASM domain (Fig. 1*b*).

To validate these predictions, both enzymes were cloned and expressed in *Escherichia coli*. After purification, they exhibited the typical brownish coloration of iron-sulfur cluster containing enzymes (Fig. 1, *c* and *d* and Fig. S2). RumMC2 proved to be more stable under *in vitro* conditions and was further characterized. After anaerobic reconstitution, the iron-sulfur cluster content of RumMC2 increased, as judged by the UV-visible spectrum (Fig. 1*d*) and iron assay, from 3.02 ± 0.14 moles of iron/mole of protein in the as-purified enzyme to 11.5 ± 0.8 moles of iron/mole of protein in the reconstituted enzyme. These results were consistent with the presence of three [4Fe-4S] clusters in RumMC2 like in most SPASM domain radical SAM enzymes characterized to date. EPR analysis of reduced RumMC2 exhibited the typical spectrum of reduced [4Fe-4S] cluster, which was well-simulated with the g-tensor values as [2.03, 1.93, 1.86] (Fig. 1*e*). This spectrum was similar to the ones reported for other SPASM domain radical SAM enzymes such as anSME (38, 40) and AlbA (33). Upon addition of SAM, the EPR spectrum was modified with novel g-tensor values of [2.03, 1.92, 1.89]. This new rhombic g-tensor is indicative of the interaction between SAM and the radical SAM [4Fe-4S] cluster as shown for pyruvate formate lyase-activating enzyme and other radical SAM enzymes (38, 40, 49). To better characterize the iron-sulfur clusters, two mutants were generated. In one mutant (A3-mutant), the three cysteine residues of the radical SAM motif (C*X*3C*X*2C) were replaced by alanine residues. In a second mutant (A7-mutant), in addition to the three cysteine residues of the C*X*3C*X*2C motif, the four cysteine residues predicted to be involved in the coordination of the second auxiliary cluster (*i.e.* AuxII; Cys^450^, Cys^453^, Cys^459^, and Cys^481^) were replaced by alanine residues. The iron content of the A3 and A7 mutants, after anaerobic reconstitution, proved to be 7.96 ± 0.12 and 5.31 ± 0.05 moles of iron/mole of protein, respectively, supporting the presence of two [4Fe-4S] clusters in the A3 mutant and a single [4Fe-4S] cluster in the A7 mutant. EPR analysis of reduced A3 mutant (Fig. 1*e*, *right panel*) exhibited the typical spectrum of reduced [4Fe-4S] clusters with g-tensor values as [2.04, 1.93, 1.88]. These data support that, in addition to the radical SAM [4Fe-4S] cluster, RumMC2 harbors additional [4Fe-4S] clusters. The A7 mutant, which was designed to contain only one auxiliary cluster (AuxI, based on the structure of anSME (46)), did not exhibit an observable EPR signal after sodium dithionite treatment. Although this might be attributed to protein instability, a recent investigation of PqqE, another SPASM domain radical SAM enzyme, by Britt and co-workers (50), has shown a similar behavior. In this study, the authors elegantly demonstrated that only the AuxII cluster is reducible by sodium dithionite whereas the AuxI cluster proved to be a very low-potential cluster.

### RumMC2 in vitro activity

**Figure 2:**
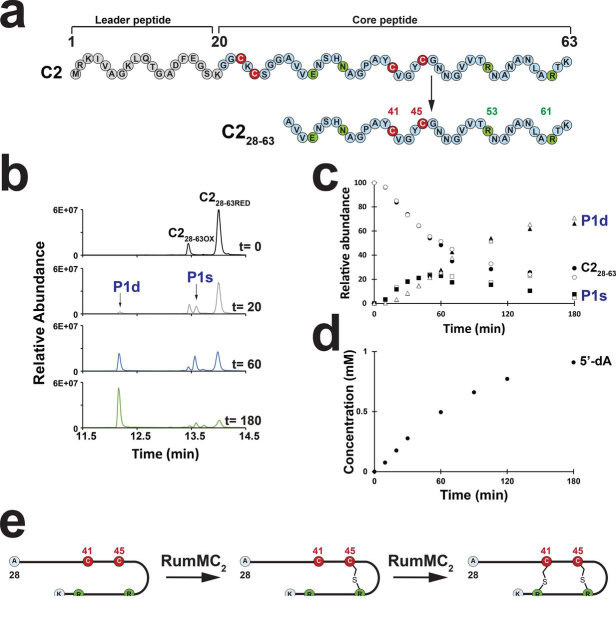
***In vitro* activity of RumMC2.***a*, sequence of the C2 peptide and the C2_28–63_ peptide used as substrate. Numbering refers to position of amino acid residues in the full-length peptide. Amino acid residues involved in thioether bridges are indicated in *red* (cysteine) and *green*. *b*, LC–MS analysis of the C2_28–63_ peptide incubated with RumMC2. The C2_28–63_ peptide eluted as oxidized (*C2_28–63_ OX*) and reduced (*C2_28-63_ RED*) forms. After 20 min of incubation, under anaerobic and reducing conditions in the presence of RumMC2, SAM, DTT, and dithionite, two peptide products (P1s and P1d) were formed. After 180 min of reaction, the P1d peptide was the major product. *c* time-course analysis of the C2_28–63_, P1s and P1d peptides formed during reaction with RumMC2. Quantification was performed using LC–MS/MS analysis. C2_28–63_ peptide (650 µm) was incubated in the presence of RumMC2 (350 µm) as described under “Experimental procedures.” Experiments were performed in duplicate. *d*, time-course analysis of 5′-dA produced during reaction with RumMC2. Quantification was performed using HPLC analysis. See “Experimental procedures” for experimental conditions. *e*, proposed model for the sequential formation of thioether bonds by RumMC2.

We recently showed that RumMC2 catalyzes the formation of thioether bonds on the C2 peptide which contains 63 amino acid residues and also on a truncated peptide called C2_28–63_ (7) (Fig. 2*a*). This peptide, which elutes as both an oxidized and reduced form under our analytical conditions ([M + 4H]^4+^ = 926.45 and 926.95, respectively) (Table S1), led to the formation of a product P1s eluting at 13.6 min when incubated in the presence of SAM, reducing agent, and reconstituted RumMC2 (Fig. 2*b* and Table S1). LC–MS/MS analysis showed that this peptide ([M + 4H]^4+^ = 926.45) contained one thioether bridge connecting the residues Arg^53^ to Cys^45^ (numbered according to the C2 peptide sequence), as judged by its mass difference with the substrate (Δ_m_ = −2 Da) and the formation of peptide fragments *y10* and *y11* −*2*, containing a dehydro-arginine (Fig. S3 and Table S2). Indeed, we and others have reported that, during the fragmentation process, C_α_-thioether bridges can open and that the amino acid residue linked to the cysteine residue is then converted to an unsaturated residue, allowing the precise location of thioether bridges (7).

After 20 min of incubation, a second product P1d ([M + 4H]^4+^ = 925.95) eluting at 12 min was detected (Fig. 2*b*). LC–MS/MS analysis showed that P1d contained two thioether bridges (Δ_m_ = −4 Da, compared with the substrate) connecting Arg^53^ to Cys^45^ and Arg^61^ to Cys^41^ as shown by the peptide fragments *y3* −*2* and *y11* −*4* (Fig. S3 and Table S2). These results were consistent with the substrate being first converted to P1s, a singly bridged species, then to P1d, a doubly bridged species.

Kinetic analysis of the reaction showed that the concentration of P1s plateaued at ∼60 min before slowly decreasing, whereas P1d accumulated over 180 min (Fig. 2*c*). At this time point, ∼500 μm of P1d containing two thioether bridges and ∼1 mm of 5′-deoxyadenosine (5′-dA) were formed (Fig. 2, *c* and *d*), supporting that one molecule of SAM is consumed per thioether bridge formed. The apparent rates of formation for P1s and P1d were 4.8 μmol min^−1^ and 3.7 μmol min^−1^, respectively, although both reactions were not performed under steady-state conditions. Because the concentration of P1s compared with the substrate was negligible at the beginning of the reaction, these results suggest either a stronger affinity for P1s or a processive mode of action for the enzyme. Collectively, these data support that thioether bridges are formed in a sequential order with formation of the Arg^53^-Cys^45^ bridge preceding formation of the Arg^61^-Cys^41^ bridge (Fig. 2*e*).

### Substrate promiscuity of RumMC2

**Figure 3:**
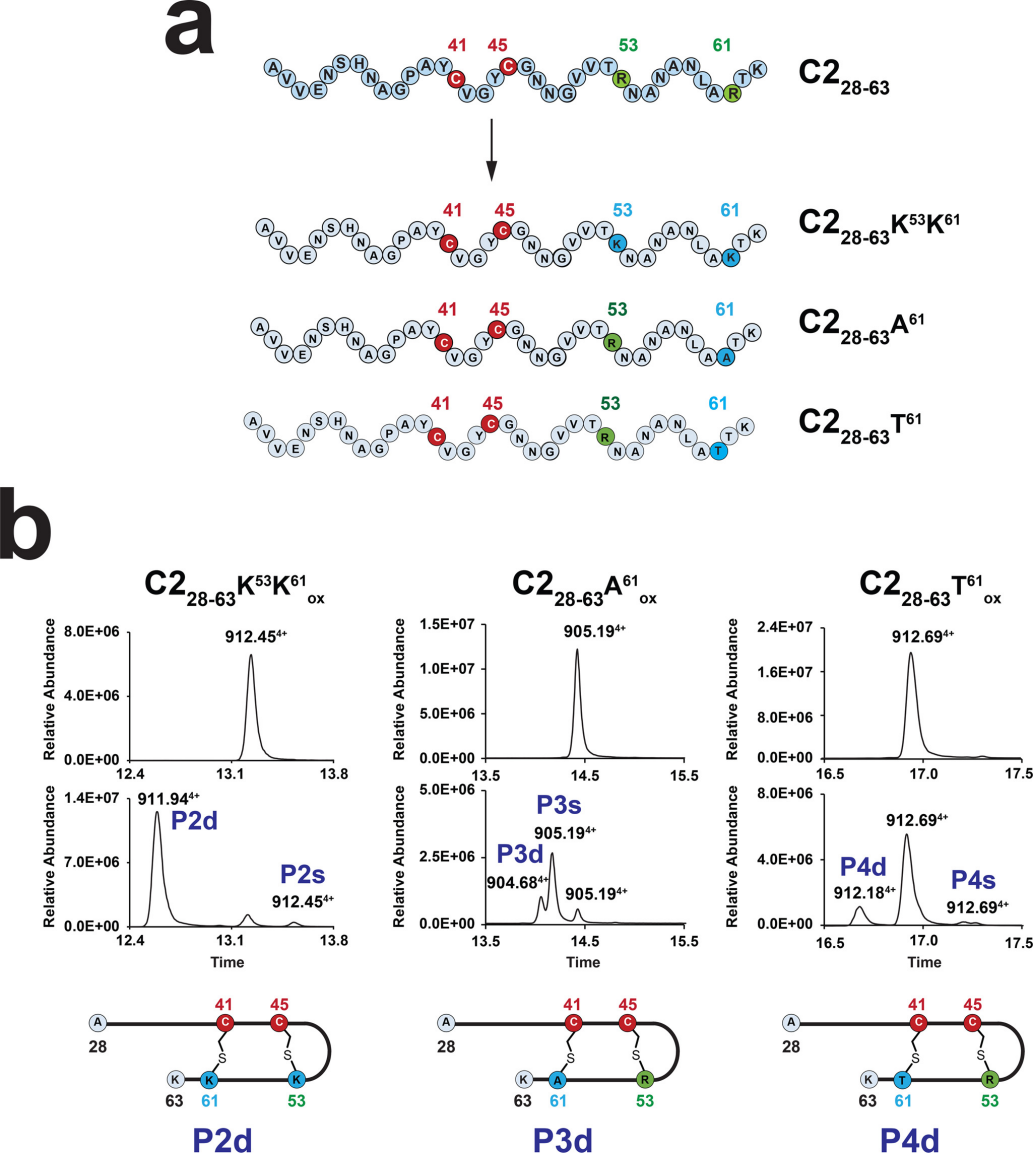
**Substrate promiscuity of RumMC2.***a*, based on the C2_28–63_ peptide, three peptides variants: C2_28–63_-K^53^K^61^, C2_28–63_-A^61^, and C2_28–63_-T^61^ were designed. Numbering refers to position of amino acid residues in C2. Amino acid residues involved in thioether bridges are indicated in *red* (cysteine) and *green*. Substituted residues are highlighted in *blue*. See Figs. S5–S7, Table S1, and Tables S3–S5 for full assignment. *b*, LC–MS analysis of the reactions performed with the peptide variants: C2_28-63_-K^53^K^61^, C2_28–63_-A^61^, and C2_28–63_-T^61^ after incubation with RumMC2. *Upper panel* T = 0 min; *Lower panel* T = 240 min. For each peptide, two products (*i.e.* species s and d) were obtained. The structures of species P2d, P3d, and P4d containing two thioether bridges are indicated below each panel. Numbers refer to *m*/*z* ratios.

To probe for the selectivity and the mechanism of RumMC2, we designed variants of the C2_28–63_ peptide in which the two arginine residues (*i.e.* Arg^53^ and Arg^61^) targeted by RumMC2 were replaced by amino acid residues with different properties (positively charged, polar and nonpolar amino acids). In a first attempt, Arg^53^ and Arg^61^ were substituted by lysine residues (Fig. 3*a*), as found in two of the five peptides encoded in the RumC operon (*i.e.* C1 and C3, Fig. S4) (6, 7). This novel peptide (C2_28–63_-K^53^K^61^) was efficiently modified by RumMC2 *in vitro* leading to the formation of two products: a singly P2s ([M + 4H]^4+^ = 912.45) and a doubly P2d ([M + 4H]^4+^ = 911.94) bridged species (Fig. 3*b*, *left panel*; Fig. S5; and Table S3). After 4 h of incubation, the doubly bridged species, containing Lys^53^-Cys^45^ and Lys^61^-Cys^41^ thioether bridges, was the major species, a result similar to the one obtained with the WT substrate.

When the substrate was modified to contain an Ala residue in position 61, (C2_28–63_-A^61^), RumMC2 catalyzed the formation of a major product (species P3s, [M + 4H]^4+^ = 905.19) containing one thioether bridge and a minor product with two thioether bridges (species P3d, [M + 4H]^4+^ = 904.68) (Fig. 3*b*, *middle panel*; Table S1, and Table S4). LC–MS/MS analysis confirmed that this latter product had, in addition to the Arg^53^-Cys^45^ thioether bridge, an Ala^61^-Cys^41^ thioether bridge (Fig. S6). Finally, substitution of Arg^61^ by the branched threonine amino acid residue resulted in a poor substrate with a low conversion level of ∼10% (Fig. 3*b*, *right panel*; Fig. S7; and Table S5). Despite this low level of conversion, the major product formed, P4d, was a doubly bridged species. Thus, whereas substitution of Arg^61^ by an Ala residue favored the accumulation of a singly bridged species, substitution by a Thr residue reduced the global yield of the reaction but led to the accumulation of doubly bridged species.

**Figure 4:**
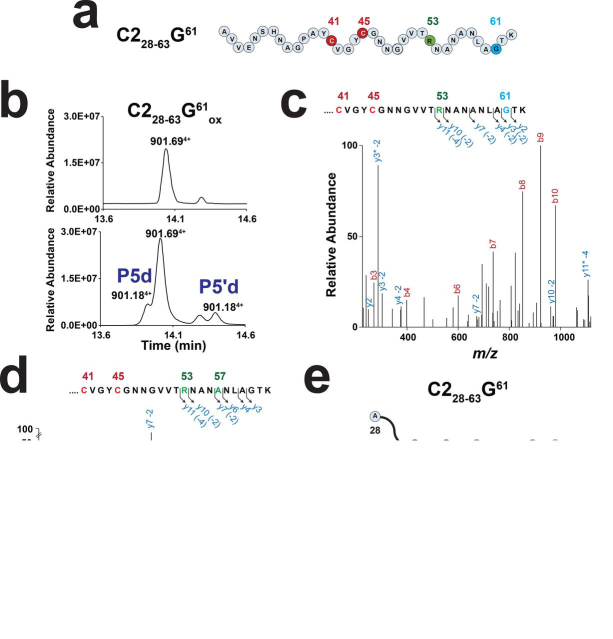
**Activity of RumMC2 on a glycine-containing peptide (C2_28–63_-G^61^).***a*, sequence of the C2_28–63_-G^61^ peptide. Numbering refers to position of amino acid residues in C2. Amino acid residues involved in thioether bridges are indicated in *red* (cysteine) and *green*. The substituted residue is highlighted in *blue*. *b*, LC–MS analyses of the C2_28–63_-G^61^ peptide before (*upper panel*) and after (*lower panel*) 240 min of incubation with RumMC2. Two new species, P5d and P5′d, with a mass of [M + 4H]^4+^ = 901.18 were produced. *c*, MS/MS analysis of P5d. Relevant ion fragments are indicated. As shown, the *y4* −2 Da, *y3* −2 Da, and *y2* ions allow to position the second thioether bridge on G^61^. *Asterisk* indicates loss of ammonia (−17.02 Da). See Table S1 and Table S6 for full assignment. *d*, MS/MS analysis of P5′d. Relevant ion fragments are indicated. As shown, the *y7* −2 Da and *y6* ion fragments allow to position the second thioether bridge on Ala^57^. *Asterisk* indicates loss of ammonia (−17.02 Da). See Table S1 and Table S6. *e*, conversion of the C2_28–63_-G^61^ peptide into two doubly bridged peptides by RumMC2.

To further probe the influence of the amino acid side chain, we designed a novel substrate containing a glycine residue (C2_28–63_-G^61^; [M + 4H]^4+^ = 901.69). Assay with this substrate led to the formation of two species, P5d and P5′d (Fig. 4, *a* and *b* and Table S1). These two species ([M + 4H]^4+^ = 901.18) had mass difference Δ_m_ = −4 Da compared with the substrate, indicating the formation of two thioether bridges. Species P5d contained in addition to the Arg^53^-Cys^45^ thioether bridge a second bridge connecting Gly^61^ to Cys^41^ (Fig. 4*c* and Table S6). Up to now, no thioether bridge involving a Gly residue had been reported in natural products (8).

The P5′d species proved to have, in addition to the Arg^53^-Cys^45^ bridge, a second thioether bridge involving Ala^57^ and Cys^41^ (Fig. 4, *d* and *e*). This result was unexpected because even when using chimeric and hybrid peptides (51), it has never been reported that sactipeptide synthases could catalyze *in vivo* or *in vitro* the formation of thioether bonds at different locations other than the natural ones.

Thus, with the exception of the Lys-to-Arg mutations (C2_28–63_-K^53^K^61^ peptide), introduction of an Ala, Thr, or Gly in position 61 decreased the reaction yield. However, even substitution by a glycine residue did not abrogate RumMC2 activity. Intriguingly, Gly mutation significantly altered the recognition of the substrate by the enzyme, leading to the introduction of an “out of frame” (Ala^57^-Cys^41^) bridge. Of note, this new bridge was located three residues away from the Arg^53^-Cys^45^ bridge, a distance identical to the one separating the two thioether bridges located in the N terminus of RumC (*i.e.* Glu^31^-Cys^24^ and Asn^35^-Cys^22^ bridges) (Fig. 1*a*).

### Substrate H-atom abstraction by RumMC2

**Figure 5:**
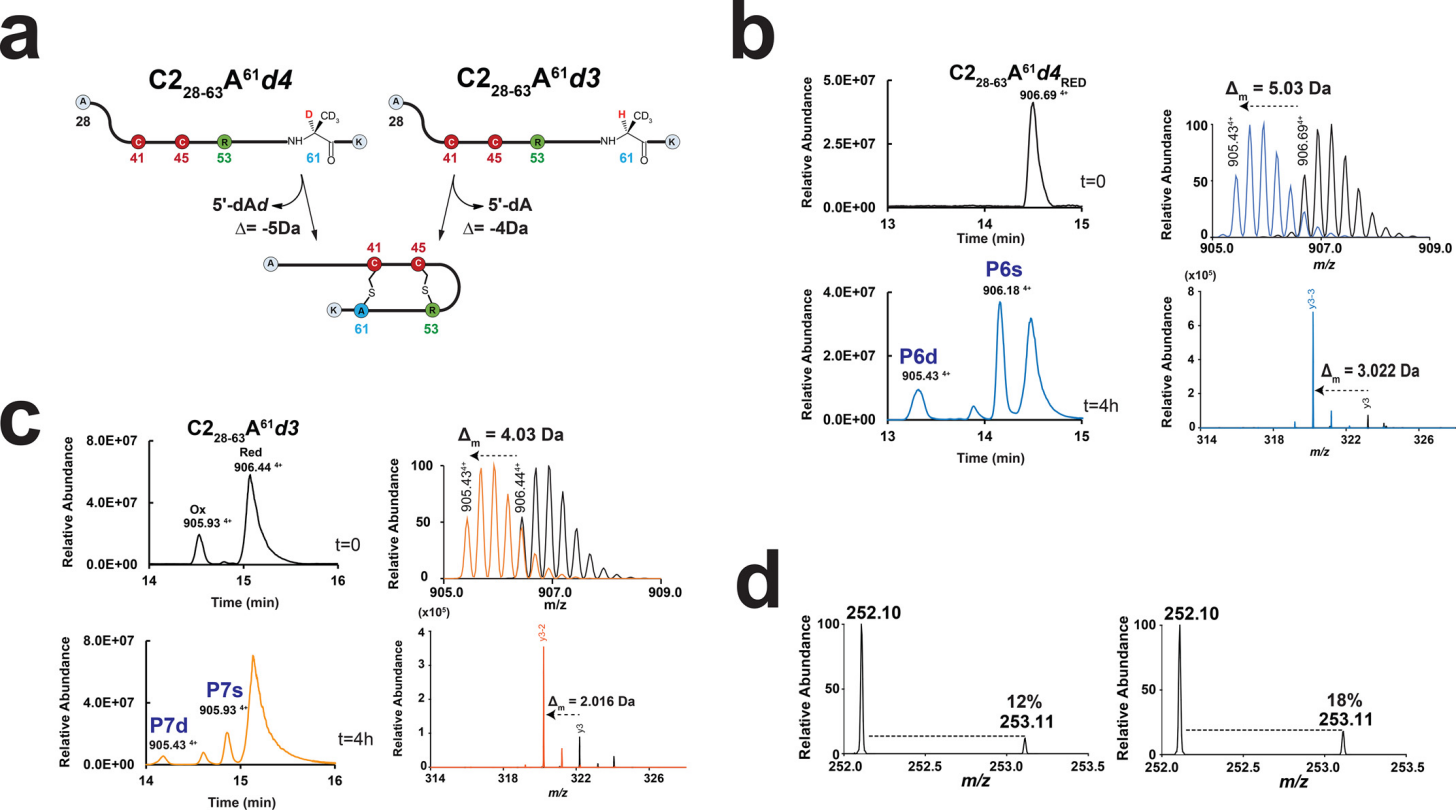
**Activity of RumMC2 on the C2_28–63_-A^61^*d3* and C2_2863_-A^61^*d4* deuterated peptides.***a*, sequence of the C2_28–63_-A^61^*d4* and C2_28–63_-A^61^*d3* peptides. The expected mass shift with the doubly bridged product (*i.e.* −4 and −5 Da, respectively) is indicated. Only with the C2_28–63_-A^61^*d4* peptide, production of 5′-dA, enriched with deuterium (*i.e.* 5′-dA*d*), is expected. *b*, LC–MS analysis of C2_28–63_-A^61^*d4* peptide before (*upper left panel*) and after 240 min of incubation with RumMC2 (*lower left panel*). Mass spectra of the substrate ([M + 4H]^4+^ = 906.69) and the doubly bridged product ([M + 4H]^4+^ = 905.43) are indicated (*upper right panel*). The relevant *y3* ion fragment originating from the product (*blue trace*; *lower right panel*) is superimposed with the *y3* ion fragment from the substrate (*black trace*; *lower right panel*). See Fig. S8, Fig. S9, Table S1 and Table S7 for full assignment. *c*, LC–MS analysis of C2_28–63_-A^61^*d3* peptide before (*upper left panel*) and after (*lower left panel*) 240 min of incubation with RumMC2. Mass spectra of the substrate ([M + 4H]^4+^ = 906.44) and the doubly bridged product ([M + 4H]^4+^ = 905.43) are indicated (*upper right panel*). The relevant *y3* ion fragment originating from the product (*orange trace*) is superimposed with the *y3* ion fragment from the substrate (*black trace*; *lower right panel*). See Table S8 for full assignment. *d*, mass spectra of 5′-dA produced in the presence of the C2_28–63_-A^61^*d3* (*left panel*) or C2_28–63_-A^61^*d4* peptide (*right panel*). Percentage indicates isotopic distribution at *m*/*z* = 253.

Having established that RumMC2, like other sactisynthases such as AlbA (32, 33, 35) and SkfB (52), exhibits substrate promiscuity and tolerates Ala substitution, we used the strategy elegantly developed by Bandarian and co-workers (53) to probe for H-atom abstraction. We synthesized two peptides: one containing a perdeuterated Ala (*i.e.* C2_28–63_-A^61^*d4* peptide) and a second one containing an Ala residue labeled only on the β-methyl moiety, in position 61 (C2_28–63_-A^61^*d3* peptide) (Fig. 5*a*). Incubation with C2_28–63_-A^61^*d4* led to the formation of two species (Fig. 5*b*). The major product (species P6s; [M + 4H]^4+^ = 906.18) had a mass shift of Δ_m_ = −2.01 Da compared with the substrate ([M + 4H]^4+^ = 906.69, Table S1), supporting the formation of one thioether bridge. The minor product (species P6d; [M + 4H]^4+^ = 905.43) had a mass shift of −3.04 Da compared with P6s, indicating the additional loss of one H- and one D-atom. LC–MS analysis confirmed the formation of the Arg^53^-Cys^45^ thioether bridge and of the Ala^61^*d4*-Cys^41^ bridge (Fig. 5*b*, Fig. S8, Fig. S9, and Table S7).

Incubation of RumMC2 with C2_28-63_-A^61^*d3* (which eluted as both an oxidized [M + 4H]^4+^ = 905.93 and reduced form [M + 4H]^4+^ = 906.44) led to the production of a singly bridged P7s ([M + 4H]^4+^ = 905.93, Table S8) and doubly bridged P7d ([M + 4H]^4+^ = 905.43) species (Fig. 5*c*). These two species, having a mass shifts of Δ_m_ = −2.02 and −4.04 Da compared with the substrate, resulted from the loss of two and four H-atoms, respectively (Fig. 5*c* and Table S8). Because the only difference between C2_28–63_-A^61^*d4* and C2_28–63_-A^61^*d3* was the presence of one deuterium on the C_α_-atom, these results unambiguously demonstrated that RumMC2 catalyzes C_α_ H-atom abstraction. They also definitively established that RumC contains C_α_-thioether bridges.

Further analysis of the 5′-dA produced during catalysis showed that, compared with the natural isotopic distribution (∼12%) (Fig. 5*d*), the 5′-dA produced in the presence of C2_28–63_-A^61^*d4* contained ∼6% isotopic enrichment at *m*/*z* = 253. This modest but significant increase validates that RumMC2 generates the 5′-dA^•^ radical to catalyze C_α_ H-atom abstraction. This ratio was also consistent with the fact that RumMC2 produced a mixture of singly P6s (∼73%) and doubly P6d (∼27%) bridged species with only the Ala^61^-Cys^41^ bridge accounting for deuterium atom abstraction (Fig. 5*b*). Futhermore, presence of low level of unlabeled substrate (<5%) also contributed to reduce the isotopic enrichment. In an attempt to improve this ratio, we synthesized a novel peptide substrate containing two perdeuterated Ala residues in positions 53 and 61 (*i.e.* C2_28–63_-A^53^-A^61^*d8*). However, this peptide was not processed by RumMC2.

### A novel mechanism for LC-MS/MS fragmentation of thioether bonds

**Figure 6:**
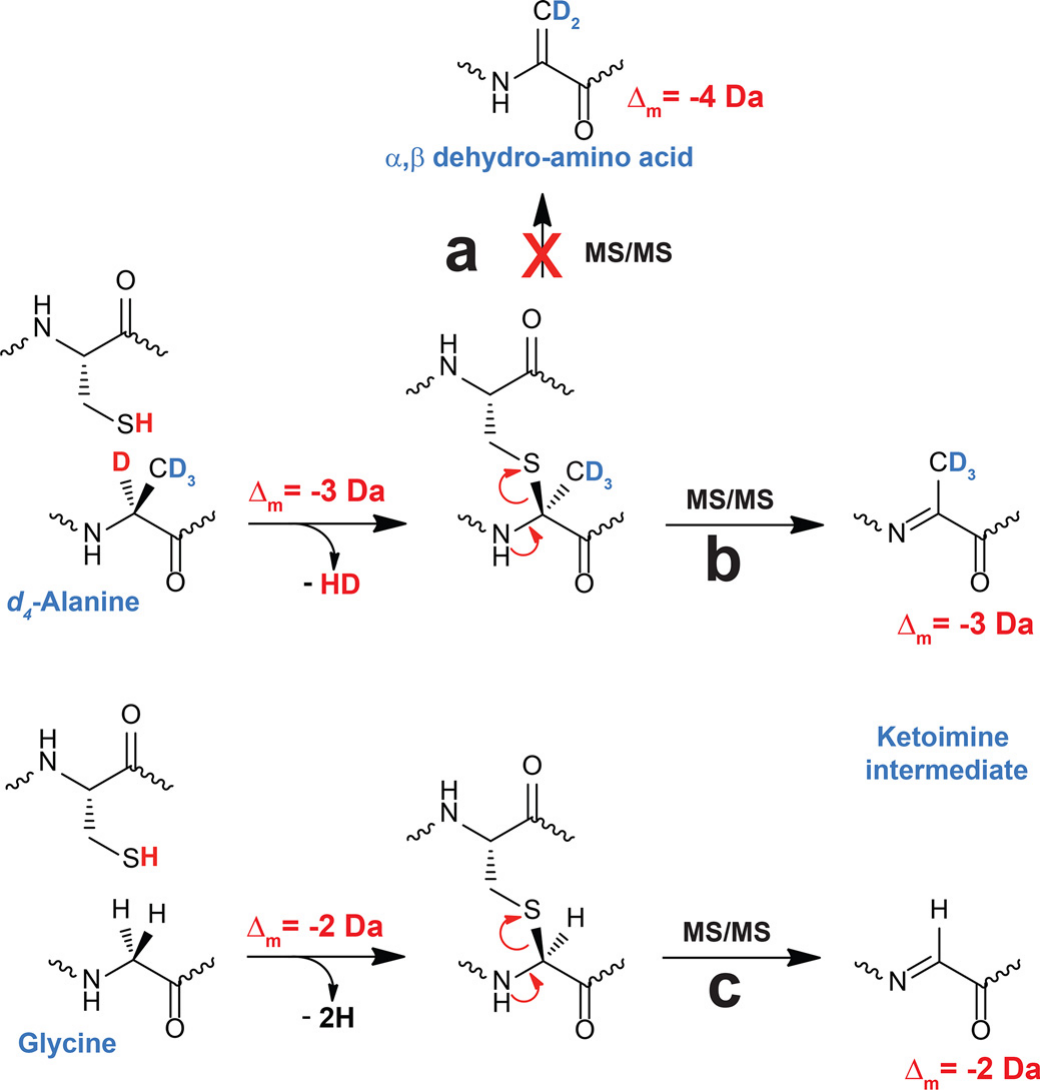
**Proposed mechanism for C_α_-thioether bond breakage during LC–MS/MS analysis.** Starting from a perdeuterated alanine residue (*d_4_*-Alanine), formation of a thioether bond leads to a mass shift of Δ_m_ = −3 Da. Thioether bond breakage (*path a*) leading to the formation of an α,β-dehydro-amino acid residue requires the elimination of a C_β_ D-atom resulting in a mass shift of Δ_m_ = −4 Da from the substrate. Experiments show that the fragmentation of thioether bridges leads exclusively to the formation of deuterated peptide fragments with a mass shift of Δ_m_ = −3 Da from the substrate. This is consistent with the formation of a ketoimine intermediate (*path b*) or an α,β-dehydro-amino acid residue following tautomerization and intramolecular deuterium migration from a ketoimine intermediate. Starting from a thioether bridge involving a glycine residue (*path c*), formation of peptide fragments with a mass shift of Δ_m_ = −2 Da is consistent with a ketoimine intermediate as previously suggested.

As explained previously, C_α_-thioether bridges are highly labile under mild voltage MS/MS analysis (7, 36). Following thioether bond opening, one cysteine residue and an α,β-dehydro-amino acid residue are formed, allowing to precisely locate the amino acid residues involved in thioether bridges (7, 54, 55). This α,β-dehydro-amino acid residue results from the loss of the C_α_ H-atom (during formation of the thioether bridge) followed by the subsequent loss of the C_β_ H-atom during mass fragmentation. Following this logic, when analyzing P6d containing a bridge with a deuterated alanine residue (Fig. 5), we should have monitored the formation of peptide fragments having lost two D-atoms (Fig. 6, *path a*). However, at odds with this hypothesis, we measured only peptide fragments having a mass shift of Δ_m_ = −3 Da (Fig. 5*b* and Fig. S9). Similarly, when using the C2_28–63_-A^61^*d3* peptide, only peptide fragments with a mass shift Δ_m_ = −2 Da were measured (Fig. 5*c*), despite the presence of three deuterium atoms on the C_β_. These experimental data showed that peptide fragments were 1 Da lighter than expected (Fig. 6, *path b*; Fig. S9; Table S7; and Table S8).

Interestingly, the P5d species containing the Gly^61^-Cys^41^ thioether bridge produced peptide fragments with a mass shift Δ_m_ = −2 Da on Gly^61^ (*i.e. y10* −2 Da to *y3* −2 Da and *y2*; Fig. 4*c*), despite the absence of a side chain. Because an α,β-dehydro-amino acid residue cannot be generated from a glycine residue, a ketoimine intermediate is the only plausible solution here (Fig. 6, *path c*).

Collectively, these results support that, unless α,β-dehydro-amino acid residues are formed by tautomerization and intramolecular H-atom migration, LC–MS/MS fragmentation of C_α_-thioether bonds leads to the formation of ketoimine intermediate.

### RRE domain of RumMC2

The role of the RRE domain (also called PqqD domain) in radical SAM enzymes was first investigated in ThnB, a sactipeptide synthase involved in thurincin H biosynthesis (56). It was shown that deletion of the RRE does not impair SAM cleavage activity but prevents the formation of thioether bonds, supporting a role for enzyme-substrate interaction. More recently, in the biosynthetic pathway of freyrasin, a RiPP containing six C_β_-thioether bonds (36), deletion of the RRE domain proved to impair freyrasin production *in vivo* but complementation *in trans* was sufficient to partially rescue enzyme activity (57).

**Figure 7:**
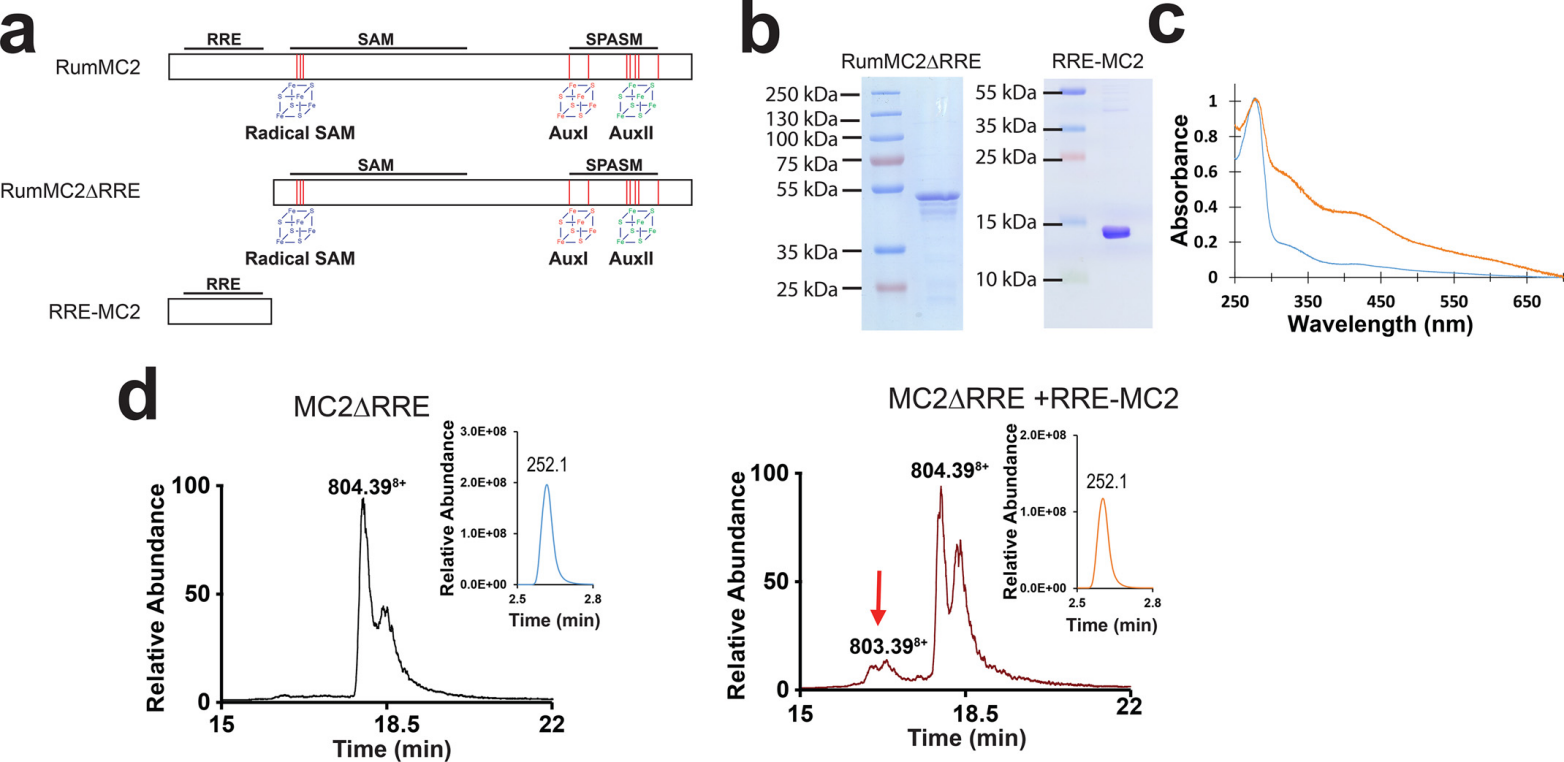
**Role of the RRE domain in the sactisynthase RumMC2.***a*, schematic representation of RumMC2 showing its three domains (RRE, SAM, and SPASM domains) and the location of the three [4Fe-4S] clusters. *b*, SDS-PAGE analysis of RumMC2ΔRRE and RRE-MC2. *c*, UV-visible spectrum of RumMC2ΔRRE before (*blue trace*) and after (*orange trace*) anaerobic iron-sulfur cluster reconstitution. *d*, LC–MS analysis of C2 incubated with RumMC2ΔRRE alone (*left panel*) or RumMC2ΔRRE in the presence of the RRE domain (RRE-MC2) (*right panel*). In *inset*, LC–MS analysis of 5′-dA produced during the reaction.

In RumMC2, we predicted that the RRE domain was located between the amino acid residues 16–94 (Fig. 1*b*). To probe its function, we generated two mutants, one deleted of the first 111 amino acid residues called RumMC2ΔRRE, and a second one containing only the N-terminal domain (*i.e.* residues 1–111) and called RRE-MC2 (Fig. 7*a*). Both mutants were expressed and purified as soluble proteins (Fig. 7*b*). The RumMC2ΔRRE mutant was reconstituted under anaerobic conditions (Fig. 7*c*) and assayed with C2 and C2_28–63_. Despite being able to cleave SAM, no modification of the substrates was evidenced. However, when the RRE domain (*i.e.* RRE-MC2) was added to the reaction mix, a small conversion (<10%) of C2 was monitored (Fig. 7*D* and Fig. S10) supporting an important role for substrate recognition. In contrast, no modification was observed with the truncated substrate C2_28–63_, under the same conditions.

## Discussion

We have recently reported that RumC is a sactipeptide containing four C_α_-thioether bridges with a unique architecture (7). These thioether bridges are critical for the antimicrobial activity of RumC against various Gram-positive bacteria, including *Clostridium* and *Bacillus* species (7). Formation of these thioether bridges is dependent on two putative and highly homologous radical SAM enzymes, RumMC1 and RumMC2, which can modify the C1 and C2 precursor peptides and truncated peptide variants. Here, we demonstrate that RumMC2 is a SPASM domain radical SAM enzyme harboring three [4Fe-4S] clusters, as shown by the presence of the conserved radical SAM motif, its UV-visible spectra, the formation of 5′-dA during catalysis (Figure 2, Figure 3, Figure 4, Figure 5*D*), and mutagenesis and EPR data (Fig. 1, *D* and *E*, Fig. S1, and Fig. S2).

In addition to the three cysteine residues involved in the coordination of the radical SAM [4Fe-4S] cluster (Cys^126^, Cys^130^, and Cys^133^), our results are consistent with the AuxI [4Fe-4S] cluster being partially coordinated by three cysteine residues (Cys^394^, Cys^413^, and Cys^462^) and the AuxII [4Fe-4S] cluster being fully coordinated by four cysteine residues (Cys^450^, Cys^453^, Cys^459^, and Cys^481^). Among radical SAM enzymes modifying peptides and proteins, several enzymes have been shown to house a fully ligated AuxI cluster involving a remote protein residue (*i.e.* Asp in PqqE or Cys in anSME and SuiB). Alternatively, the fourth ligand of the AuxI cluster could be a cysteine residue from the peptide itself (34). Further studies will be required to discern between both possibilities in RumMC2.

Based on kinetic data, we have shown here that formation of each thioether bond requires the cleavage of one molecule of SAM, supporting that SAM is a co-substrate (Fig. 2). Up to now, partly because of the difficulties to handle such hydrophobic peptides, kinetic data have been only reported for AlbA (32). However, in this case, a strong uncoupling between SAM cleavage and thioether bond formation was measured (32). Our data also establish that RumMC2 catalyzes thioether bridge formation in a defined order with the Arg^53^-Cys^45^ bridge being installed before the Arg^61^-Cys^41^ bridge (Fig. 2*E*). Interestingly, mutation of the residue in position 61 affects the formation of not only the second bridge but also the first one (Fig. 3). Collectively, these results favor a processive mode of action for RumMC2, but a combination between cooperativity and processivity cannot be excluded. So far, the only processive radical SAM enzyme described was PoyD, a radical SAM epimerase (26) that introduces posttranslational modifications in a strictly ordered manner.

Although sactipeptide synthases have been reported to catalyze the formation of a breadth of thioether bridges, the formation of bridges involving a glycine residue was unknown until recently. Investigation of the C2_28–63_-G^61^ peptide showed that, contrary to other peptide variants, RumMC2 catalyzed the formation of two doubly bridged species: P5d and P5′d (Fig. 4). Whereas P5d contained the Arg^53^-Cys^45^ and the Gly^61^-Cys^41^ bridges, the P5′d species exhibited a nonnatural Ala^57^-Cys^41^ bridge. To the best of our knowledge, the synthesis of out of frame posttranslational modifications has never been reported for other sactisynthases. Only the radical SAM epimerase PoyD, when using a synthetic substrate, installs epimerizations in nonnatural positions (26). Interestingly, with this peptide variant, RumMC2 modified a residue (*i.e.* Ala^57^) located three residues apart from Arg^53^, the same distance as between the two N-terminal thioether bridges (*i.e.* Glu^31^ and Asn^35^) (Fig. 1*A*). This result suggests that not only the side chain of the amino acid residue but also other factors likely govern the correct installation of posttranslational modifications in sactipeptides.

LC–MS/MS analysis of deuterated peptides containing thioether bridges revealed discrepancies between the expected and measured mass of peptide fragments (Fig. 6). For instance, analysis of a peptide containing a thioether bridge with a perdeuterated Ala residue led to the formation of peptide fragments with a mass shift Δ_m_ = −3 Da. This result, which implies the loss of an H- and a D-atom, is likely explained by the formation of a ketoimine intermediate. This ketoimine intermediate might tautomerize in an α,β-dehydro-amino acid residue but, to be consistent with the masses observed, this would require an intramolecular D-atom migration. However, when analyzing the peptide containing the Gly^61^-Cys^41^ bridge, we measured peptide fragments with a mass difference Δ_m_ = −2 Da compared with the unmodified peptide. Because of the absence of an amino acid side chain, only the formation of a ketoimine intermediate can be invoked here. Currently, the mechanism of sactisynthases and other radical SAM enzymes installing thioether bridges is not fully resolved. Notably, after substrate H-atom abstraction and formation of a carbon-centered radical, the nature of the reactive substrate intermediate has yet to be determined. We proposed in an earlier study that the substrate radical could undergo rearrangement into a ketoimine intermediate, allowing the facile addition to a cysteine thiolate from the peptide and formation of a thioether bridge (32). Our MS results show that the intermediacy of such species is compatible with thioether bridge chemistry.

To conclude, in recent years, radical SAM enzymes have been shown to play a major role in RiPP biosynthesis (8). Notably, they catalyze the formation of a wide diversity of thioether bridges. Our data show that RumMC2 is SPASM domain radical SAM enzyme with a likely processive mode of action. Although a growing number of RiPP biosynthetic pathways are investigated, it is likely that an increasing number of processive posttranslational modification enzymes will be uncovered. Such processivity could be connected to the function of the RRE domain, which is critical for thioether bridge installation, even in peptides lacking the leader sequence. Finally, in addition to novel insights into thioether bridge biosynthesis, our study opens new routes for the synthesis of designer RiPPs using radical SAM enzymes.

## Experimental procedures

### Reagents

All Fmoc-amino acid residues and *O*-benzotriazol-1-yl-*N,N,N′,N′*-tetramethyluronium hexafluorophosphate were purchased from Christof Senn Laboratories (Dielsdorf, Switzerland) or Novabiochem (Darmstadt, Germany). Preloaded 4-hydroxymethyl-phenoxymethyl-copolystyrene-1%-divinylbenzene resins were obtained from Life Technologies (Villebon sur Yvette, France). *N,N*-Diisopropylethylamine, piperidine, TFA, triisopropylsilane, *tert-*butylmethylether were supplied from Sigma-Aldrich (Saint-Quentin-Fallavier, France). Dimethylformamide (DMF) was from Biosolve (Dieuze, France). Dichloromethane (DCM) and acetonitrile were from Fisher Scientific (Illkirch, France).

### Cloning, expression, and purification of RumMC2, RumMC1 and the RumMC2A7 mutant

The cloning, expression, and purification of the radical SAM enzyme RumMC2 were performed as described previously (7). Briefly, optimized gene was synthesized by Life Technologies (Thermo Fisher Scientifc, GeneArt®) and ligated in a pET-28a plasmid (pET28a-RumMC2) and transformed in *E. coli* BL21 (DE3) (Life Technologies). The proteins were expressed and purified under aerobic conditions by affinity chromatography (Strep-Tag) and flash-frozen in liquid nitrogen (7), and purity was assessed by SDS-PAGE. The same strategy was used for the cloning, expression and purification of RumMC1 and the RumMC2A7 mutant.

### Cloning, expression, and purification of RumMC2A3 mutant

The mutant was obtained by two site-directed mutagenesis, the first one using the pET28a-RumMC2 plasmid as a template and the primers 5′-GACCGAACAGGCTAATATGCGT-3′ and 5′-ACGCATATTAGCCTGTTCGGTC-3′ to mutate Cys^126^ residue to alanine. After sequencing, the plasmid containing the mutation C126A was used as a template for a second site-directed mutagenesis with the primers 5′-GCGTGCTCGTTATGCCATCTAT-3′ and 5′-ATAGATGGCATAACGAGCACGC-3′ to mutate Cys^130^ and Cys^133^ residues to alanine. After sequencing, the plasmid encoding for the triple alanine variant was transformed in *E. coli* BL21 (DE3) star cells (Life Technologies). Expression and purification were performed like for the WT enzyme.

### Cloning, expression, and purification of RumMC2ΔRRE and RRE-MC2 domains

RuMC2ΔRRE mutant was obtained by site-directed mutagenesis using the pET28a-RumMC2 plasmid as a template and the primers 5′-CATCACAGCAGCGGCCGTTATGATCTGCAG-3′ and 5′-CTGCAGATCATAACGGCCGCTGCTGTGATG-3′ to remove the first 111 codons. After verification of the correct deletion, the protein was expressed and purified as the WT enzyme. Protein purity was assayed on a 12% SDS–PAGE.

RRE-MC2 mutant was obtained by site-directed mutagenesis, using as the template the pET28a-RumMC2 plasmid and the primers 5′-CCCTGCGTGATCTGCTGTAATATGATCTGCAGCA-3′ and 5′-TGCTGCAGATCATATTACAGCAGATCACGCAGGG-3′ to insert a stop codon after the 111th codon. After verification of the correct deletion, the protein was expressed and purified as described for the wild-type enzyme. Protein purity was assayed on a 12% SDS–PAGE.

### Peptide synthesis

Peptides C2_28–63_-A^61^*d3* and C2_28–63_-A^61^*d4* were synthesized by Fmoc solid phase methodology on a Liberty microwave assisted automated peptide synthesizer (CEM, Saclay, France) using the standard manufacturer's procedures at 0.1 mmol scale as previously described (58). Briefly, all Fmoc-amino acids (0.5 mmol, 5 eq.) were coupled on preloaded Fmoc-Lys(Boc)-4-hydroxymethyl-phenoxymethyl-copolystyrene-1%-divinylbenzene resins resin by *in situ* activation with *O*-benzotriazol-1-yl-*N*,*N*,*N*′,*N*′-tetramethyluronium hexafluorophosphate (0.5 mmol, 5 eq.) and *N*,*N*-Diisopropylethylamine (1 mmol, 10 eq.), and Fmoc removal was performed with a 20% piperidine solution in DMF. After completion of the chain assembly, the peptide was deprotected and cleaved from the resin by adding 10 ml of the mixture TFA/triisopropylsilane/H_2_O (9.5:0.25:0.25) for 180 min at room temperature. After filtration, crude peptide was washed thrice by precipitation in *tert-*butylmethylether followed by centrifugation (4500 rpm, 15 min). The synthetic peptide was purified by reversed-phase HPLC on a 21.2 × 250 mm Jupiter C_18_ (5 μm, 300 Å) column (Phenomenex, Le Pecq, France) using a linear gradient (10–50% or 10–40% over 45 min) of acetonitrile/TFA (99.9:0.1) at a flow rate of 10 ml/min. The purified peptide was then characterized by MALDI-TOF MS on an UltrafleXtreme (Bruker, Strasbourg, France) in the reflector mode using α-cyano-4-hydroxycinnamic acid as a matrix. Analytical reversed-phase HPLC, performed on a 4.6 × 250 mm Jupiter C_18_ (5 μm, 300 Å) column (Phenomenex), indicated that the purity of the peptide was >99.9%.

Other peptides were synthesized by Proteogenix and resuspended in DMSO. Peptides: C2_28–63_, AVVENSHNAGPAYCVGYCGNNGVVTRNANANLARTK; C2_28–63_-A^61^, AVVENSHNAGPAYAVGYCGNNGVVTRNANANLAATK; C2_28–63_T^61^, AVVENSHNAGPAYCVGYAGNNGVVTRNANANLATTK; C2_28–63_G^61^, AVVENSHNAGPAYAVGYAGNNGVVTRNANANLAGTK; and C2_28–63_-K^53^K^61^, AVVENSHNAGPAYCVGYCGNNGVVTKNANANLAKTK.

### Iron-sulfur cluster reconstitution and iron content titration

Iron-sulfur cluster reconstitution was performed as described previously (7). Iron content titration was performed using a solution of Mohr's salt (for calibration curve) and protein samples with a known concentration. Both were mixed with 100 μl of 1% HCl and incubated at 80 °C for 10 min. After cooling and centrifugation, 500 μl of 7.5% ammonium acetate, 100 μl of 4% ascorbic acid, 100 μl of 2.5% SDS, and 100 μl of 1.5% Ferene were successively added. After centrifugation, the absorbance at 593 nm was measured for all samples. Based on the calibration curve, the iron content of the proteins was determined.

### UV-visible spectroscopy

UV-visible spectra of proteins were recorded on JASCO spectrophotometer.

### Enzymatic assays

All the assays were performed in an anaerobic chamber at 24 °C. Freshly prepared reconstituted protein (350 μm) was used for activity assays. All the reagents were freshly prepared and suspended in H_2_O. SAM (Sigma-Aldrich), substrate, and sodium dithionite (Sigma-Aldrich) were added successively to a final concentration of 2 mm, 650 μm, and 3 mm, respectively. Reactions were quenched by adding 0.1% formic acid for LC–MS analysis and 0.1% TFA for HPLC analysis.

### HPLC analysis and purification

Reactions were analyzed using an Eclipse C18 plus column (2 × 50 mm, 1.8 μm, 100 Å, Agilent) by loading 10–20 μl of each sample diluted 10 times in 0.1% v/v formic acid. Elution was performed at a flow rate of 0.3 ml/min using an acetonitrile gradient between 10–30% v/v of acetonitrile 80% v/v and formic acid 0.1% v/v. Peptide UV-detection was performed at 215 nm.

### LC–MS analysis

Samples were analyzed without prior purification of the peptide using an Ultimate 3000 nanoHPLC and a Vanquish UHPLC systems by LC–MS connected to a Q Exactive Focus mass spectrometer (Thermo Fisher Scientific). Acetonitrile gradients between 10–30% and 15–25% in formic acid (0.1%) were used. Mass analysis was performed at a resolution of 35,000 (*m*/*z* 200) with a MS range of 500–1300 and MS/MS analysis. Data were processed using Xtract tools included in the Freestyle software suite version 1.3 (Thermo Fisher Scientific). All daughter ions were verified and annotated manually.

### EPR analysis

X-band EPR spectra were recorded on a Bruker ELEXSYS 500 spectrometer equipped with a Bruker ER 4116DM X-band resonator, an Oxford Instrument continuous flow ESR 900 cryostat, and an Oxford ITC 503 temperature control system.

Conditions: Microwave frequency = 9.636 GHz, microwave power = 1.0 milliwatt, modulation amplitude = 8 Gauss, modulation frequency = 100 KHz, Gain = 40 db, temperature =10 K.

Simulations were done using the Bruker software XSophe.

## Data availability

All the data supporting the findings of this study are available within this article and in the supporting information.

10.13039/100010663EC | H2020 | H2020 Priority Excellent Science | H2020 European Research Council (ERC) (617053) to Olivier Berteau
10.13039/501100001665Agence Nationale de la Recherche (ANR) (ANR-17-CE11-0014) to Olivier Berteau

## References

[1] Benjdia A., Guillot A., Ruffié P., Leprince J., Berteau O. (2017). Post-translational modification of ribosomally synthesized peptides by a radical SAM epimerase in *Bacillus subtilis*. Nat. Chem.

[2] Balskus E.P. (2018). The human microbiome. ACS Infect. Dis.

[3] Chittim C.L., Irwin S.M., Balskus E.P. (2018). Deciphering human gut microbiota-nutrient interactions: A role for biochemistry. Biochemistry.

[4] Cohen L.J., Han S., Huang Y.H., Brady S.F. (2018). Identification of the colicin V bacteriocin gene cluster by functional screening of a human microbiome metagenomic library. ACS Infect. Dis.

[5] Chu J., Vila-Farres X., Inoyama D., Gallardo-Macias R., Jaskowski M., Satish S., Freundlich J.S., Brady S.F. (2018). Human microbiome inspired antibiotics with improved β-lactam synergy against MDR *Staphylococcus aureus*. ACS Infect Dis.

[6] Pujol A., Crost E.H., Simon G., Barbe V., Vallenet D., Gomez A., Fons M. (2011). Characterization and distribution of the gene cluster encoding RumC, an anti-Clostridium perfringens bacteriocin produced in the gut. FEMS Microbiol. Ecol.

[7] Balty C., Guillot A., Fradale L., Brewee C., Boulay M., Kubiak X., Benjdia A., Berteau O. (2019). Ruminococcin C, an anti-clostridial sactipeptide produced by a prominent member of the human microbiota *Ruminococcus gnavus*. J. Biol. Chem.

[8] Benjdia A., Balty C., Berteau O. (2017). Radical SAM enzymes in the biosynthesis of ribosomally synthesized and post-translationally modified peptides (RiPPs). Front Chem.

[9] Mahanta N., Hudson G.A., Mitchell D.A. (2017). Radical *S*-adenosylmethionine enzymes involved in RiPP biosynthesis. Biochemistry.

[10] Benjdia A., Decamps L., Guillot A., Kubiak X., Ruffié P., Sandström C., Berteau O. (2017). Insights into the catalysis of a lysine-tryptophan bond in bacterial peptides by a SPASM domain radical *S*-adenosylmethionine (SAM) peptide cyclase. J. Biol. Chem.

[11] Imai Y., Meyer K.J., Iinishi A., Favre-Godal Q., Green R., Manuse S., Caboni M., Mori M., Niles S., Ghiglieri M., Honrao C., Ma X., Guo J.J., Makriyannis A., Linares-Otoya L. (2019). A new antibiotic selectively kills Gram-negative pathogens. Nature.

[12] Ongey E.L., Giessmann R.T., Fons M., Rappsilber J., Adrian L., Neubauer P. (2018). Heterologous biosynthesis, modifications and structural characterization of Ruminococcin-A, a lanthipeptide from the gut bacterium *Ruminococcus gnavus* E1, in *Escherichia coli*. Front. Microbiol.

[13] Crost E.H., Ajandouz E.H., Villard C., Geraert P.A., Puigserver A., Fons M. (2011). Ruminococcin C, a new anti-Clostridium perfringens bacteriocin produced in the gut by the commensal bacterium *Ruminococcus gnavus* E1. Biochimie.

[14] Chiumento S., Roblin C., Kieffer-Jaquinod S., Tachon S., Leprètre C., Basset C., Aditiyarini D., Olleik H., Nicoletti C., Bornet O., Iranzo O., Maresca M., Hardré R., Fons M., Giardina T. (2019). Ruminococcin C, a promising antibiotic produced by a human gut symbiont. Sci. Adv.

[15] Ortega M.A., van der Donk W.A. (2016). New insights into the biosynthetic logic of ribosomally synthesized and post-translationally modified peptide natural products. Cell Chem Biol.

[16] Hetrick K.J., van der Donk W.A. (2017). Ribosomally synthesized and post-translationally modified peptide natural product discovery in the genomic era. Curr. Opin. Chem. Biol.

[17] Horitani M., Shisler K., Broderick W.E., Hutcheson R.U., Duschene K.S., Marts A.R., Hoffman B.M., Broderick J.B. (2016). Radical SAM catalysis via an organometallic intermediate with an Fe-[5′-C]-deoxyadenosyl bond. Science.

[18] Broderick J.B., Duffus B.R., Duschene K.S., Shepard E.M. (2014). Radical S-adenosylmethionine enzymes. Chem. Rev.

[19] Duschene K.S., Veneziano S.E., Silver S.C., Broderick J.B. (2009). Control of radical chemistry in the AdoMet radical enzymes. Curr. Opin. Chem. Biol.

[20] Decamps L., Philmus B., Benjdia A., White R., Begley T.P., Berteau O. (2012). Biosynthesis of F0, precursor of the F420 cofactor, requires a unique two radical-SAM domain enzyme and tyrosine as substrate. J. Am. Chem. Soc.

[21] Philmus B., Decamps L., Berteau O., Begley T.P. (2015). Biosynthetic versatility and coordinated action of 5′-deoxyadenosyl radicals in deazaflavin biosynthesis. J. Am. Chem. Soc.

[22] Benjdia A., Pierre S., Gherasim C., Guillot A., Carmona M., Amara P., Banerjee R., Berteau O. (2015). The thiostrepton A tryptophan methyltransferase TsrM catalyses a cob(II)alamin-dependent methyl transfer reaction. Nat. Commun.

[23] Pierre S., Guillot A., Benjdia A., Sandström C., Langella P., Berteau O. (2012). Thiostrepton tryptophan methyltransferase expands the chemistry of radical SAM enzymes. Nat. Chem. Biol.

[24] Parent A., Guillot A., Benjdia A., Chartier G., Leprince J., Berteau O. (2016). The B12-radical SAM enzyme PoyC catalyzes valine Cβ-methylation during polytheonamide biosynthesis. J. Am. Chem. Soc.

[25] Popp P.F., Benjdia A., Strahl H., Berteau O., Mascher T. (2020). The epipeptide YydF intrinsically triggers the cell envelope stress response of *Bacillus subtilis* and causes severe membrane perturbations. Front. Microbiol.

[26] Parent A., Benjdia A., Guillot A., Kubiak X., Balty C., Lefranc B., Leprince J., Berteau O. (2018). Mechanistic investigations of PoyD, a radical *S*-adenosyl-l-methionine enzyme catalyzing iterative and directional epimerizations in polytheonamide A biosynthesis. J. Am. Chem. Soc.

[27] Freeman M.F., Gurgui C., Helf M.J., Morinaka B.I., Uria A.R., Oldham N.J., Sahl H.G., Matsunaga S., Piel J. (2012). Metagenome mining reveals polytheonamides as posttranslationally modified ribosomal peptides. Science.

[28] Khaliullin B., Ayikpoe R., Tuttle M., Latham J.A. (2017). Mechanistic elucidation of the mycofactocin-biosynthetic radical *S*-adenosylmethionine protein, MftC. J. Biol. Chem.

[29] Bhandari D.M., Fedoseyenko D., Begley T.P. (2016). Tryptophan lyase (NosL): A cornucopia of 5′-deoxyadenosyl radical mediated transformations. J. Am. Chem. Soc.

[30] Bhandari D.M., Fedoseyenko D., Begley T.P. (2018). Mechanistic studies on tryptophan lyase (NosL): Identification of cyanide as a reaction product. J. Am. Chem. Soc.

[31] Schramma K.R., Bushin L.B., Seyedsayamdost M.R. (2015). Structure and biosynthesis of a macrocyclic peptide containing an unprecedented lysine-to-tryptophan crosslink. Nat. Chem.

[32] Benjdia A., Guillot A., Lefranc B., Vaudry H., Leprince J., Berteau O. (2016). Thioether bond formation by SPASM domain radical SAM enzymes: Cα H-atom abstraction in subtilosin A biosynthesis. Chem. Commun.

[33] Flühe L., Knappe T.A., Gattner M.J., Schäfer A., Burghaus O., Linne U., Marahiel M.A. (2012). The radical SAM enzyme AlbA catalyzes thioether bond formation in subtilosin A. Nat. Chem. Biol.

[34] Grove T.L., Himes P.M., Hwang S., Yumerefendi H., Bonanno J.B., Kuhlman B., Almo S.C., Bowers A.A. (2017). Structural insights into thioether bond formation in the biosynthesis of sactipeptides. J. Am. Chem. Soc.

[35] Himes P.M., Allen S.E., Hwang S., Bowers A.A. (2016). Production of sactipeptides in *Escherichia coli*: Probing the substrate promiscuity of subtilosin A biosynthesis. ACS Chem. Biol.

[36] Hudson G.A., Burkhart B.J., DiCaprio A.J., Schwalen C., Kille B., Pogorelov T.V., Mitchell D.A. (2019). Bioinformatic mapping of radical SAM-dependent RiPPs identifies new Cα, Cβ, and Cγ-linked thioether-containing peptides. J. Am. Chem. Soc.

[37] Benjdia A., Leprince J., Guillot A., Vaudry H., Rabot S., Berteau O. (2007). Anaerobic sulfatase-maturating enzymes: Radical SAM enzymes able to catalyze in vitro sulfatase post-translational modification. J. Am. Chem. Soc.

[38] Benjdia A., Subramanian S., Leprince J., Vaudry H., Johnson M.K., Berteau O. (2008). Anaerobic sulfatase-maturating enzymes, first dual substrate radical *S*-adenosylmethionine enzymes. J. Biol. Chem.

[39] Haft D.H., Basu M.K. (2011). Biological systems discovery in silico: Radical *S*-adenosylmethionine protein families and their target peptides for posttranslational modification. J. Bacteriol.

[40] Benjdia A., Subramanian S., Leprince J., Vaudry H., Johnson M.K., Berteau O. (2010). Anaerobic sulfatase-maturating enzyme–a mechanistic link with glycyl radical-activating enzymes?. FEBS J.

[41] Grell T.A., Goldman P.J., Drennan C.L. (2015). SPASM and twitch domains in *S*-adenosylmethionine (SAM) radical enzymes. J. Biol. Chem.

[42] Burkhart B.J., Hudson G.A., Dunbar K.L., Mitchell D.A. (2015). A prevalent peptide-binding domain guides ribosomal natural product biosynthesis. Nat. Chem. Biol.

[43] Berteau O., Guillot A., Benjdia A., Rabot S. (2006). A new type of bacterial sulfatase reveals a novel maturation pathway in prokaryotes. J. Biol. Chem.

[44] Benjdia A., Dehò G., Rabot S., Berteau O. (2007). First evidences for a third sulfatase maturation system in prokaryotes from *E. coli* aslB and ydeM deletion mutants. FEBS Lett.

[45] Benjdia A., Leprince J., Sandström C., Vaudry H., Berteau O. (2009). Mechanistic investigations of anaerobic sulfatase-maturating enzyme: Direct Cβ H-atom abstraction catalyzed by a radical AdoMet enzyme. J. Am. Chem. Soc.

[46] Goldman P.J., Grove T.L., Sites L.A., McLaughlin M.I., Booker S.J., Drennan C.L. (2013). X-ray structure of an AdoMet radical activase reveals an anaerobic solution for formylglycine posttranslational modification. Proc. Natl. Acad. Sci. U. S. A.

[47] Davis K.M., Schramma K.R., Hansen W.A., Bacik J.P., Khare S.D., Seyedsayamdost M.R., Ando N. (2017). Structures of the peptide-modifying radical SAM enzyme SuiB elucidate the basis of substrate recognition. Proc. Natl. Acad. Sci. U. S. A.

[48] Barr I., Stich T.A., Gizzi A.S., Grove T.L., Bonanno J.B., Latham J.A., Chung T., Wilmot C.M., Britt R.D., Almo S.C., Klinman J.P. (2018). X-ray and EPR characterization of the auxiliary Fe-S clusters in the radical SAM enzyme PqqE. Biochemistry.

[49] Yang H., McDaniel E.C., Impano S., Byer A.S., Jodts R.J., Yokoyama K., Broderick W.E., Broderick J.B., Hoffman B.M. (2019). The elusive 5′-deoxyadenosyl radical: Captured and characterized by electron paramagnetic resonance and electron nuclear double resonance spectroscopies. J. Am. Chem. Soc.

[50] Tao L., Zhu W., Klinman J.P., Britt R.D. (2019). Electron paramagnetic resonance spectroscopic identification of the Fe-S clusters in the SPASM domain-containing radical SAM enzyme PqqE. Biochemistry.

[51] Burkhart B.J., Kakkar N., Hudson G.A., van der Donk W.A., Mitchell D.A. (2017). Chimeric leader peptides for the generation of non-natural hybrid RiPP products. ACS Cent. Sci.

[52] Flühe L., Burghaus O., Wieckowski B.M., Giessen T.W., Linne U., Marahiel M.A. (2013). Two [4Fe-4S] clusters containing radical SAM enzyme SkfB catalyze thioether bond formation during the maturation of the sporulation killing factor. J. Am. Chem. Soc.

[53] Bruender N.A., Bandarian V. (2016). SkfB abstracts a hydrogen atom from Cα on SkfA to initiate thioether cross-link formation. Biochemistry.

[54] Sit C.S., van Belkum M.J., McKay R.T., Worobo R.W., Vederas J.C. (2011). The 3D solution structure of thurincin H, a bacteriocin with four sulfur to α-carbon crosslinks. Angew. Chem. Int. Ed. Engl.

[55] Kawulka K.E., Sprules T., Diaper C.M., Whittal R.M., McKay R.T., Mercier P., Zuber P., Vederas J.C. (2004). Structure of subtilosin A, a cyclic antimicrobial peptide from *Bacillus subtilis* with unusual sulfur to α-carbon cross-links: Formation and reduction of α-thio-α-amino acid derivatives. Biochemistry.

[56] Wieckowski B.M., Hegemann J.D., Mielcarek A., Boss L., Burghaus O., Marahiel M.A. (2015). The PqqD homologous domain of the radical SAM enzyme ThnB is required for thioether bond formation during thurincin H maturation. FEBS Lett.

[57] Precord T.W., Mahanta N., Mitchell D.A. (2019). Reconstitution and substrate specificity of the thioether-forming radical *S*-adenosylmethionine enzyme in freyrasin biosynthesis. ACS Chem Biol.

[58] Touchard A., Aili S.R., Téné N., Barassé V., Klopp C., Dejean A., Kini R.M., Mrinalini, Coquet L., Jouenne T., Lefranc B., Leprince J., Escoubas P., Nicholson G.M., Treilhou M., Bonnafé E. (2020). Venom peptide repertoire of the European Myrmicine ant *Manica rubida*: Identification of insecticidal toxins. J. Proteome Res.

