# Supplementary material for: Biosynthesis of the sactipeptide Ruminococcin C by the human microbiome: Mechanistic insights into thioether bond formation by radical SAM enzymes
Source: J Biol Chem. 2021 Jan 13;295(49):16665–77. doi: 10.1074/jbc.RA120.015371 (PMC8188230; doi:10.1074/jbc.RA120.015371)
Supplement: Supplementary file 1 [file mmc1.pdf]

Supporting Information for:

Biosynthesis of the sactipeptide Ruminococcin C by the human microbiome: Mechanistic insights into thioether bond formation by radical SAM enzymes

Clémence Balty[1], Alain Guillot[1], Laura Fradale[1], Clémence Brewee[1], Benjamin Lefranc[2], Christian Herrero[3], Corine Sandström[4], Jérôme Leprince[2], Olivier Berteau[1]* & Alhosna Benjdia[1]*

[1] Université Paris-Saclay, INRAE, AgroParisTech, Micalis Institute, ChemSyBio, 78350 Jouy-en-Josas, France
[2] Université de Normandie-Rouen, INSERM U1239, PRIMACEN, 76000 Rouen, France
[3] Université Paris-Saclay, CNRS, ICMMO, 91405, Orsay, France
[4] Department of Molecular Sciences, Uppsala BioCenter, Swedish University of Agricultural Sciences, P.O. Box 7015, SE-750 07 Uppsala, Sweden

* : Corresponding authors

Figure S1 - Sequence alignment of RumMC1 & RumMC2 with other SPASM-domain radical SAM enzymes. 2
Figure S2 – SDS-PAGE analysis and UV-visible spectrum of RumMC1 before (pink trace) and after (red trace) iron-sulfur cluster reconstitution. 3
Figure S3 – LC-MS analysis of the reaction performed with the $C2_{28\text{-}63}$ peptide 4
Figure S4 - Sequence of the C1 to C5 peptides encoded by the RumC operon 5
Figure S5 - MS/MS spectrum of the P2d peptide obtained after incubation with RumMC2 6
Figure S6 - MS/MS spectrum of the P3d peptide obtained after incubation with RumMC2 7
Figure S7 – MS/MS spectrum of the P4d peptide obtained after incubation with RumMC2 7
Figure S8 – MS/MS spectrum of the P6s peptide 8
Figure S9 – MS/MS spectrum of the P6d peptide 8
Figure S10 – MS/MS analysis of the C2 peptide after incubation in the presence of RumMC2ΔRRE and RRE-MC2. 9
Table S1 – Peptides used in this study and their theoretical and observed (obs) masses 10
Table S2 - Theoretical mass fragments of peptide $C2_{28\text{-}63}$ $[M+H]^+$ 11
Table S3 - Theoretical mass fragments of peptide $C2_{28\text{-}63}K^{53}K^{61}$ $[M+H]^+$ 12
Table S4 - Theoretical mass fragments of peptide $C2_{28\text{-}63}A^{61}$ $[M+H]^+$ 13
Table S5 - Theoretical mass fragments of peptide $C2_{28\text{-}63}T^{61}$ $[M+H]^+$ 14
Table S6 - Theoretical mass fragments of peptide $C2_{28\text{-}63}G^{61}$ $[M+H]^+$ 15
Table S7 - Theoretical mass fragments of peptide $C2_{28\text{-}63}A^{61}$*d4* 16
Table S8 - Theoretical Mass fragments of peptide $C2_{28\text{-}63}A^{61}$*d3* 17

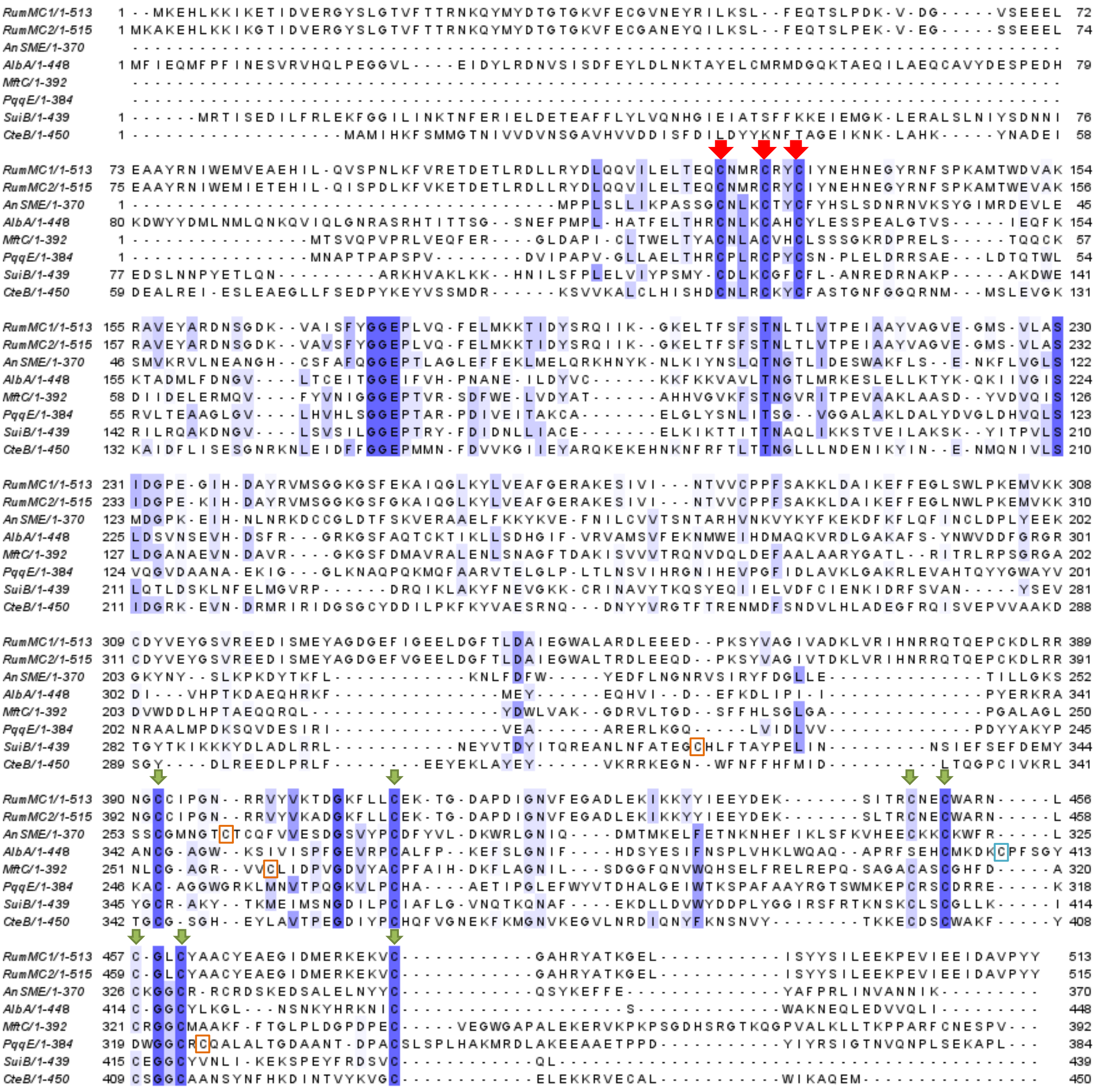

Figure S1 - Sequence alignment of RumMC1 & RumMC2 with other SPASM-domain radical SAM enzymes.

Sequences were aligned using Jalview (1). Blue highlighting is proportional to the conservation of amino acids between protein sequences. The canonical CX_3CX_2C motif is indicated by red arrows and the seven cysteine residues implicated in the SPASM motif are indicated by green arrows. For AlbA, a cysteine residue tentatively proposed to be involved in the coordination of the AuxII [4Fe-4S] cluster is squared in blue. The eighth cysteine residue, involved in the complete coordination of the AuxI [4Fe-4S] cluster in AnSME, MftC, PqqE and SuiB is squared in orange.

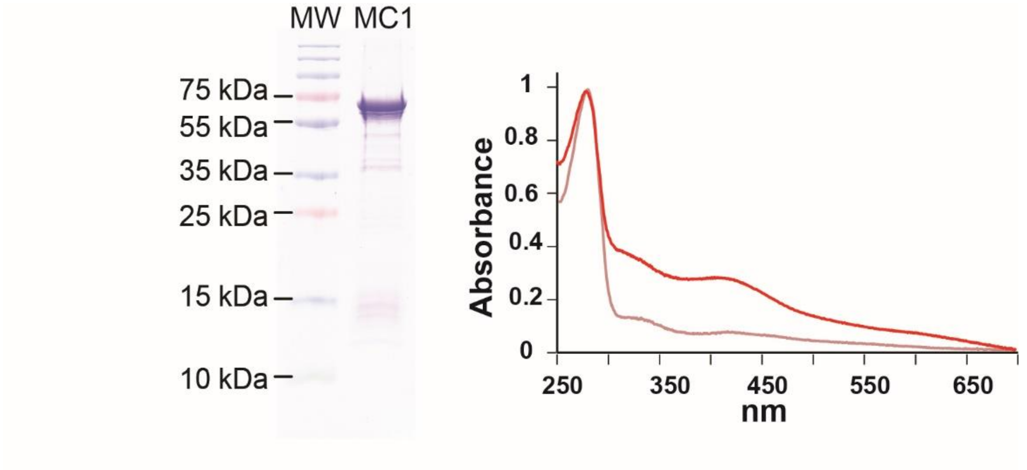

Figure S2 – SDS-PAGE analysis and UV-visible spectrum of RumMC1 before (pink trace) and after (red trace) iron-sulfur cluster reconstitution.

A

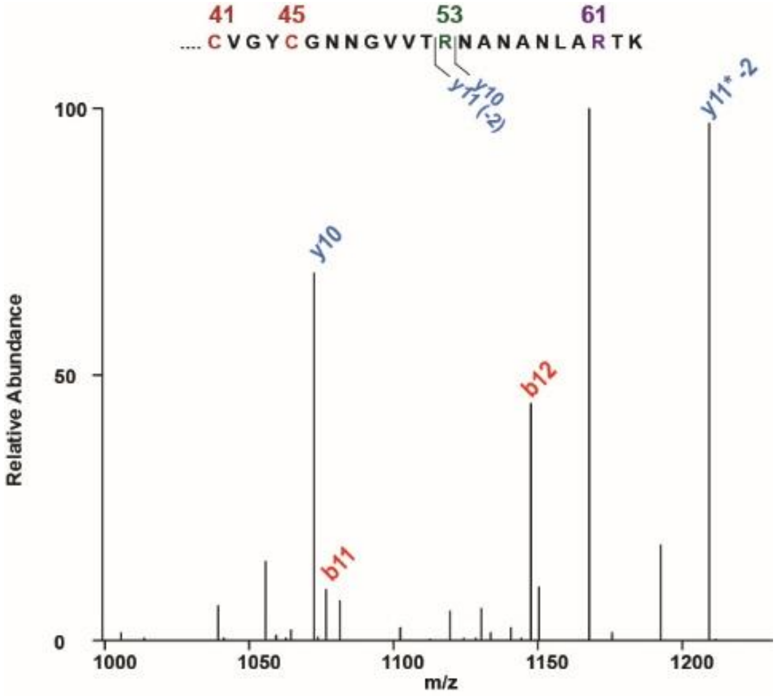

B

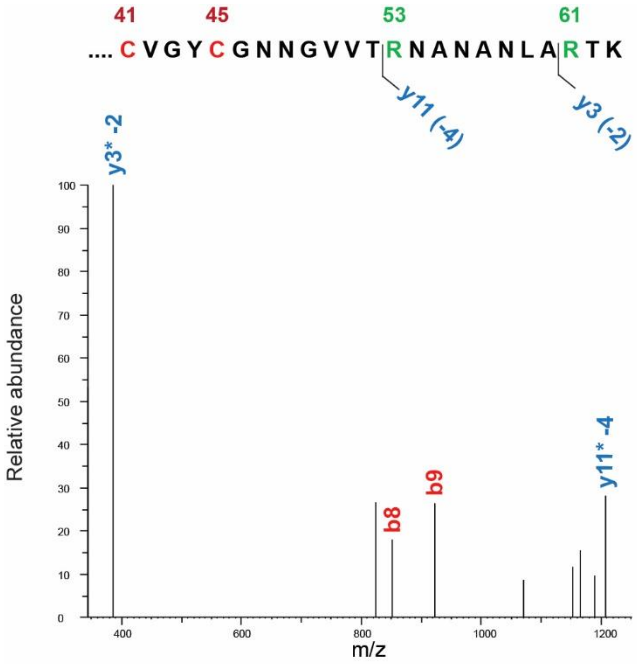

Figure S3 – LC-MS analysis of the reaction performed with the C2$_{28-63}$ peptide

(**A**) **MS/MS analysis of P1s.** Relevant peptide fragments are indicated showing that **P1s** contains a Cys^{45}-to- Arg^{53} thioether bond. (**B**) **MS/MS analysis of P1d.** Relevant peptide fragments are indicated showing that **P1d** contains a Cys^{41}-to- Arg^{61} thioether bond in addition to the Cys^{45}-to- Arg^{53} thioether bond (*see table S2* for complete assignment). * indicates loss of ammonia (-17.02 Da).

```
C1  MRKIVAGKLQTGADFEGSKWGCVCSGSTAVANSHNAGPAYCVGYCGNNGVVTRNANANVAKTA
C2  MRKIVAGKLQTGADFEGSKGGCKCSGGAVVENSHNAGPAYCVGYCGNNGVVTRNANANLARTK
C3  MKLVETKTTKTGTNFEGNRAGCICNGTVAVANSHNAGPAYCVGYCGNSGVVTRNANANVAKTA
C4  MRLVQSKRIATGFNFEGSKAGCVCSGTVAVANSHNAGPAYCVGYCGNNGEVTRNANYNIARRS
C5  MKLVTSKTMKTGTNFEGNKAGCICSGSVAVANSHNAGPAYCVGYCGNNGAVTRNANANLARTA
```

Figure S4 - Sequence of the C1 to C5 peptides encoded by the RumC operon

Arginine and lysine residues are highlighted in red and green respectively. Conserved cysteine residues are highlighted in blue.

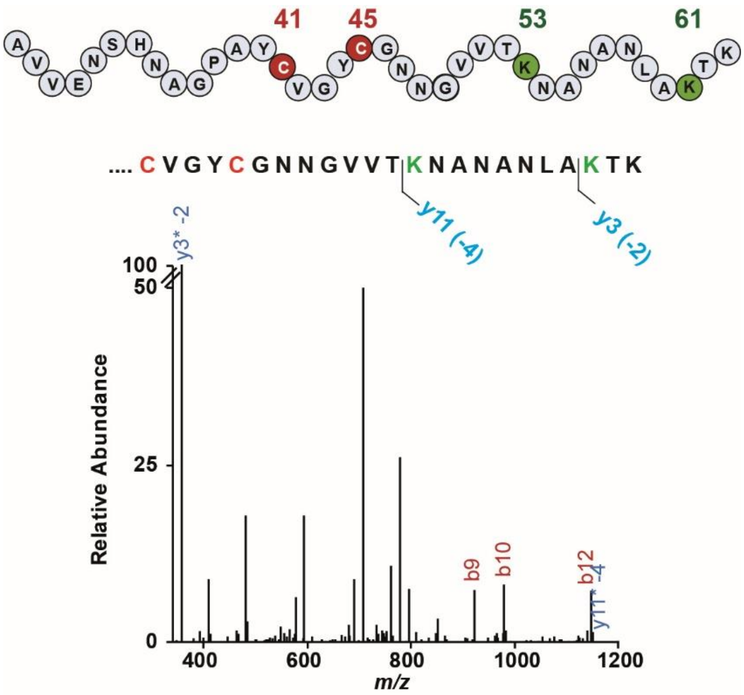

Figure S5 - MS/MS spectrum of the P2d peptide obtained after incubation with RumMC2

Relevant peptide fragments are indicated confirming that this peptide contains two thioether bridges (*i.e.* Lys^{53}-Cys^{45} and Lys^{61}- Cys^{41} bridges) (*see table S3* for complete assignment). * indicates loss of ammonia (-17.02 Da).

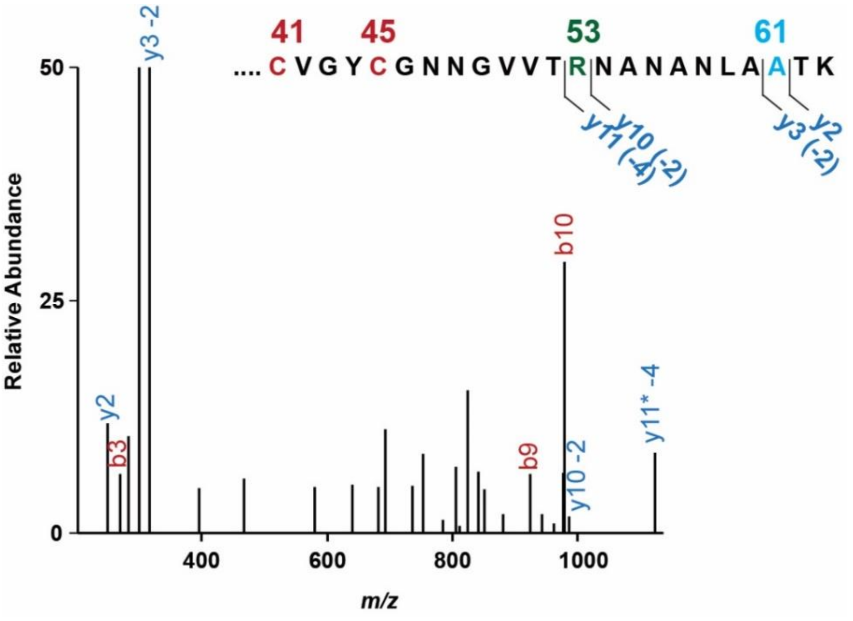

Figure S6 - MS/MS spectrum of the P3d peptide obtained after incubation with RumMC2

Relevant peptide fragments are indicated showing that this peptide contains two thioether bridges (*i.e.* Arg^{53}-Cys^{45} and Ala^{61}-Cys^{41} bridges) (*see table S4* for complete assignment). * indicates loss of ammonia (-17.02 Da).

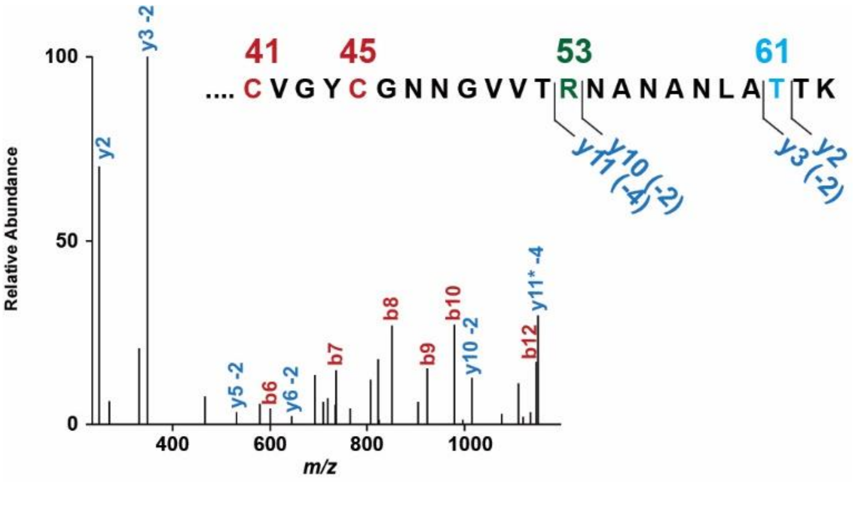

Figure S7 – MS/MS spectrum of the P4d peptide obtained after incubation with RumMC2

Relevant peptide fragments are indicated showing that this peptide contains two thioether bridges (*i.e.* Arg^{53}-Cys^{45} and Thr^{61}-Cys^{41} bridges) (*see table S5* for complete assignment). * indicates loss of ammonia (-17.02 Da).

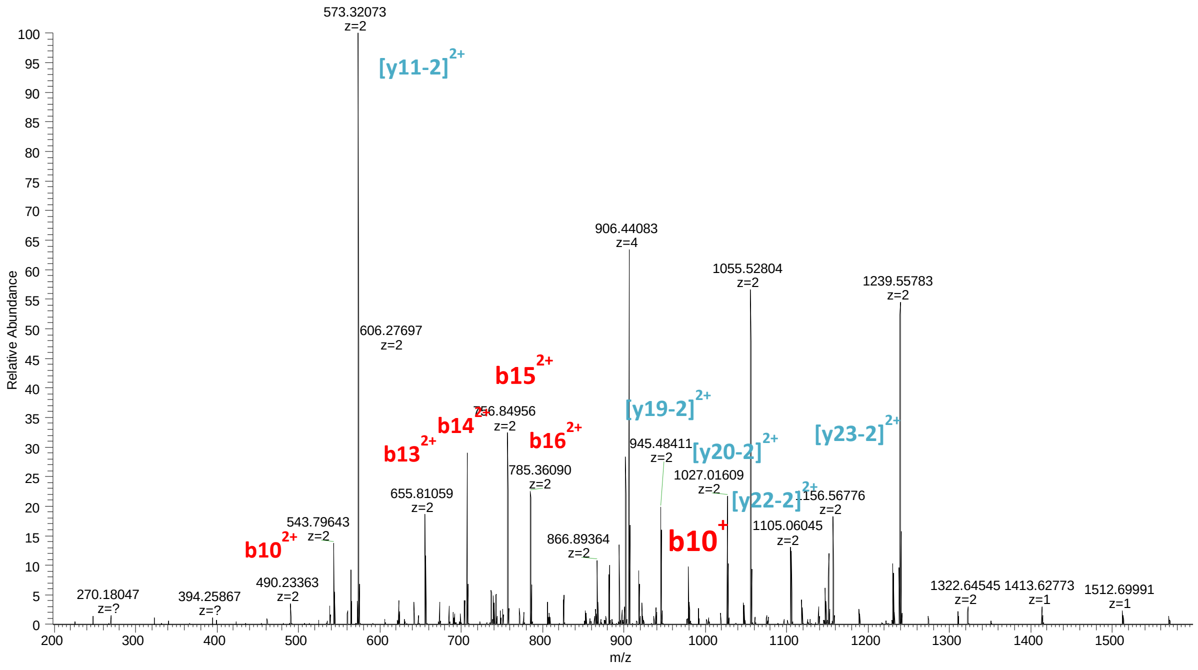

Figure S8 – MS/MS spectrum of the P6s peptide

See table S7 for complete assignment

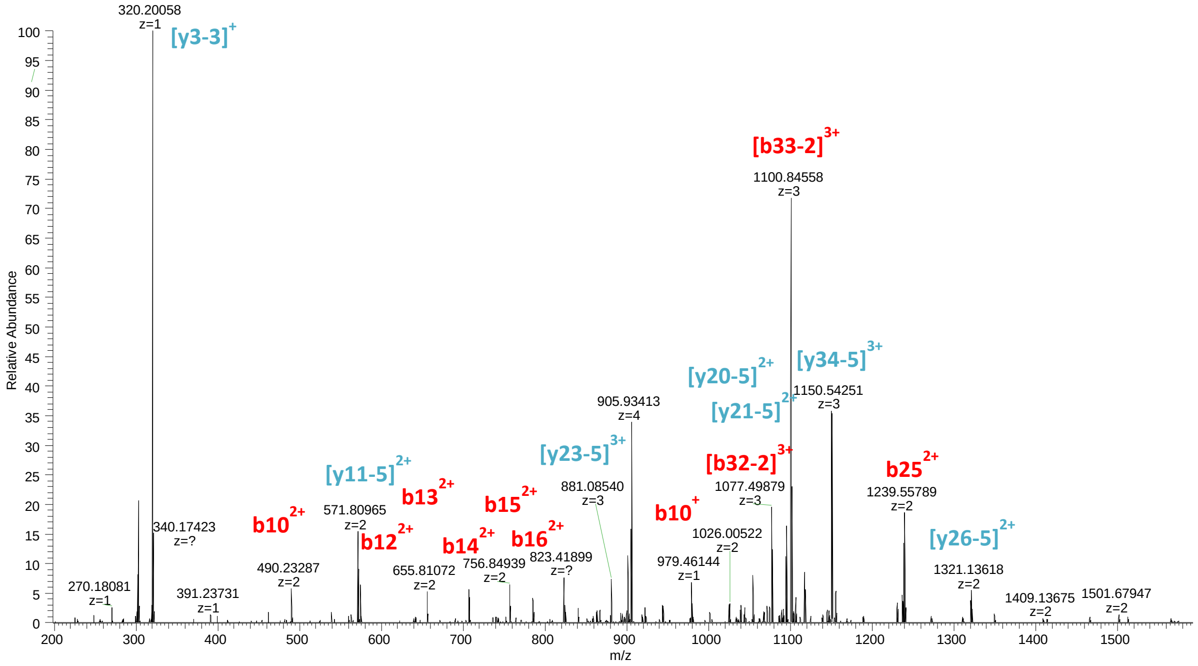

Figure S9 – MS/MS spectrum of the P6d peptide

See table S7 for complete assignment

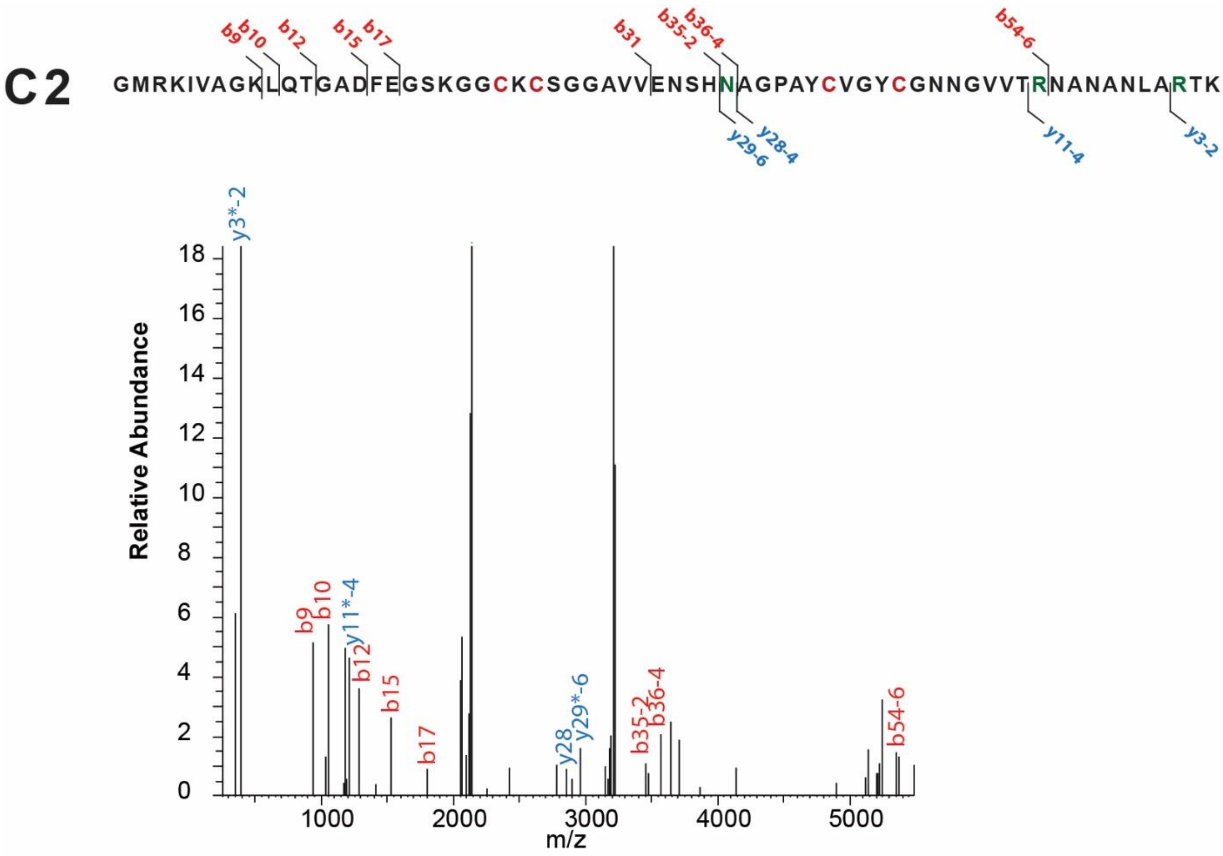

Figure S10 – MS/MS analysis of the C2 peptide after incubation in the presence of RumMC2ΔRRE and RRE-MC2.

C2 (250 µM) was incubated for 4 hours with RumMC2ΔRRE (250 µM), RRE-MC2 (250 µM), SAM (2 mM) and sodium dithionite (3 mM). * indicates loss of ammonia (-17.02 Da).

Table S1 – Peptides used in this study and their theoretical and observed (obs) masses

Peptide	Formula	$[M+4H]^{4+}$	$[M+4H]^{4+}_{obs}$
**$C2_{28-63}$**	C154 H246 N52 O51 S2	926.9497	926.9533
**$C2_{28-63}$ -2H**	C154 H244 N52 O51 S2	926.4457	926.4463
**$C2_{28-63}$ -4H**	C154 H242 N52 O51 S2	925.9419	925.9443
**$C2_{28-63}K^{53}K^{61}$**	C154 H246 N48 O51 S2	912.9466	912.9545
**$C2_{28-63}K^{53}K^{61}$ -2H**	C154 H244 N48 O51 S2	912.4427	912.4449
**$C2_{28-63}K^{53}K^{61}$ -4H**	C154 H242 N48 O51 S2	911.9388	91.9415
**$C2_{28-63}$A61**	C151 H239 N49 O51 S2	905.6837	905.6848
**$C2_{28-63}$A61 -2H**	C151 H237 N49 O51 S2	905.1797	905.1831
**$C2_{28-63}$A61 -4H**	C151 H235 N49 O51 S2	904.6759	904.6893
**$C2_{28-63}T^{61}$**	C152 H241 N49 O52 S2	913.1863	913.1920
**$C2_{28-63}T^{61}$- 2H**	C152 H239 N49 O52 S2	912.6824	912.6920
**$C2_{28-63}T^{61}$- 4H**	C152 H237 N49 O52 S2	912.1785	912.1822
**$C2_{28-63}$G61**	C150 H237 N49 O51 S2	902.1798	902.1845
**$C2_{28-63}$G61 -2H**	C150 H237 N49 O51 S2	901.6758	901.6862
**$C2_{28-63}$G61 -4H**	C150 H237 N49 O51 S2	901.1719	901.1770

Peptide	Formula	$[M+4H]^{4+}$	$[M+4H]^{4+obs}$
**$C2_{28-63}$*d3***	C151 H236 N49 O51 S2 D3	906.4384	906.4409
**$C2_{28-63}$*d3* -2H**	C151 H234 N49 O51 S2 D3	905.9344	905.9334
**$C2_{28-63}$*d3* -4H**	C151 H232 N49 O51 S2 D3	905.4306	905.4329

Peptide	Formula	$[M+4H]^{4+}$	$[M+4H]^{4+obs}$
**$C2_{28-63}$*d4***	C151 H235 N49 O51 S2 D4	906.6900	906.6928
**$C2_{28-63}$*d4* -2H**	C151 H233 N49 O51 S2 D4	906.1860	906.1890
**$C2_{28-63}$*d4* -4H**	C151 H231 N49 O51 S2 D4	905.4306	905.4344

Table S2 - Theoretical mass fragments of peptide C2$_{28\text{-}63}$ [M+H]$^{+}$

			b	*y*	
A	28	1	72.04444	3704.77691	36
V	29	2	171.11285	3633.73979	35
V	30	3	270.18126	3534.67138	34
E	31	4	399.22386	3435.60297	33
N	32	5	513.26678	3306.56037	32
S	33	6	600.29881	3192.51745	31
H	34	7	737.35772	3105.48542	30
N	35	8	851.40065	2968.42651	29
A	36	9	922.43776	2854.38358	28
G	37	10	979.45923	2783.34647	27
P	38	11	1076.51199	2726.325	26
A	39	12	1147.5491	2629.27224	25
Y	40	13	1310.61243	2558.23513	24
C	41	14	1413.62162	2395.1718	23
V	42	15	1512.69003	2292.16261	22
G	43	16	1569.71149	2193.0942	21
Y	44	17	1732.77482	2136.07273	20
C	45	18	1835.78401	1973.00941	19
G	46	19	1892.80547	1870.00022	18
N	47	20	2006.8484	1812.97876	17
N	48	21	2120.89133	1698.93583	16
G	49	22	2177.91279	1584.8929	15
V	50	23	2276.9812	1527.87144	14
V	51	24	2376.04962	1428.80303	13
T	52	25	2477.0973	1329.73461	12
R	53	26	2633.19841	1228.68693	11
N	54	27	2747.24133	1072.58582	10
A	55	28	2818.27845	958.5429	9
N	56	29	2932.32137	887.50578	8
A	57	30	3003.35849	773.46286	7
N	58	31	3117.40141	702.42574	6
L	59	32	3230.48548	588.38281	5
A	60	33	3301.52259	475.29875	4
R	61	34	3457.6237	404.26164	3
T	62	35	3558.67138	248.16053	2
K	63	36	3686.76634	147.11285	1

Table S3 - Theoretical mass fragments of peptide $C2_{28\text{-}63}K^{53}K^{61}$ $[M+H]^+$

			b	*y*	
A	28	1	72.04444	3648.76461	36
V	29	2	171.11285	3577.7275	35
V	30	3	270.18126	3478.65908	34
E	31	4	399.22386	3379.59067	33
N	32	5	513.26678	3250.54808	32
S	33	6	600.29881	3136.50515	31
H	34	7	737.35772	3049.47312	30
N	35	8	851.40065	2912.41421	29
A	36	9	922.43776	2798.37128	28
G	37	10	979.45923	2727.33417	27
P	38	11	1076.51199	2670.31271	26
A	39	12	1147.5491	2573.25994	25
Y	40	13	1310.61243	2502.22283	24
C	41	14	1413.62162	2339.1595	23
V	42	15	1512.69003	2236.15032	22
G	43	16	1569.71149	2137.0819	21
Y	44	17	1732.77482	2080.06044	20
C	45	18	1835.78401	1916.99711	19
G	46	19	1892.80547	1813.98793	18
N	47	20	2006.8484	1756.96646	17
N	48	21	2120.89133	1642.92353	16
G	49	22	2177.91279	1528.88061	15
V	50	23	2276.9812	1471.85914	14
V	51	24	2376.04962	1372.79073	13
T	52	25	2477.0973	1273.72232	12
K	53	26	2605.19226	1172.67464	11
N	54	27	2719.23518	1044.57968	10
A	55	28	2790.2723	930.53675	9
N	56	29	2904.31523	859.49963	8
A	57	30	2975.35234	745.45671	7
N	58	31	3089.39527	674.41959	6
L	59	32	3202.47933	560.37667	5
A	60	33	3273.51644	447.2926	4
K	61	34	3401.61141	376.25549	3
T	62	35	3502.65908	248.16053	2
K	63	36	3630.75405	147.11285	1

Table S4 - Theoretical mass fragments of peptide C2$_{28\text{-}63}$A^{61} [M+H]$^{+}$

			b	*y*	
A	28	1	72.04444	3619.71291	36
V	29	2	171.11285	3548.6758	35
V	30	3	270.18126	3449.60738	34
E	31	4	399.22386	3350.53897	33
N	32	5	513.26678	3221.49638	32
S	33	6	600.29881	3107.45345	31
H	34	7	737.35772	3020.42142	30
N	35	8	851.40065	2883.36251	29
A	36	9	922.43776	2769.31958	28
G	37	10	979.45923	2698.28247	27
P	38	11	1076.51199	2641.26101	26
A	39	12	1147.5491	2544.20824	25
Y	40	13	1310.61243	2473.17113	24
C	41	14	1413.62162	2310.1078	23
V	42	15	1512.69003	2207.09861	22
G	43	16	1569.71149	2108.0302	21
Y	44	17	1732.77482	2051.00874	20
C	45	18	1835.78401	1887.94541	19
G	46	19	1892.80547	1784.93622	18
N	47	20	2006.8484	1727.91476	17
N	48	21	2120.89133	1613.87183	16
G	49	22	2177.91279	1499.82891	15
V	50	23	2276.9812	1442.80744	14
V	51	24	2376.04962	1343.73903	13
T	52	25	2477.0973	1244.67062	12
R	53	26	2633.19841	1143.62294	11
N	54	27	2747.24133	987.52183	10
A	55	28	2818.27845	873.4789	9
N	56	29	2932.32137	802.44179	8
A	57	30	3003.35849	688.39886	7
N	58	31	3117.40141	617.36174	6
L	59	32	3230.48548	503.31882	5
A	60	33	3301.52259	390.23475	4
A	61	34	3372.55971	319.19764	3
T	62	35	3473.60738	248.16053	2
K	63	36	3601.70235	147.11285	1

Table S5 - Theoretical mass fragments of peptide $C2_{28\text{-}63}T^{61}$ $[M+H]^{+}$

			b	*y*	
A	28	1	72.04444	3649.72348	36
V	29	2	171.11285	3578.68636	35
V	30	3	270.18126	3479.61795	34
E	31	4	399.22386	3380.54953	33
N	32	5	513.26678	3251.50694	32
S	33	6	600.29881	3137.46401	31
H	34	7	737.35772	3050.43199	30
N	35	8	851.40065	2913.37307	29
A	36	9	922.43776	2799.33015	28
G	37	10	979.45923	2728.29303	27
P	38	11	1076.51199	2671.27157	26
A	39	12	1147.5491	2574.21881	25
Y	40	13	1310.61243	2503.18169	24
C	41	14	1413.62162	2340.11836	23
V	42	15	1512.69003	2237.10918	22
G	43	16	1569.71149	2138.04077	21
Y	44	17	1732.77482	2081.0193	20
C	45	18	1835.78401	1917.95597	19
G	46	19	1892.80547	1814.94679	18
N	47	20	2006.8484	1757.92533	17
N	48	21	2120.89133	1643.8824	16
G	49	22	2177.91279	1529.83947	15
V	50	23	2276.9812	1472.81801	14
V	51	24	2376.04962	1373.74959	13
T	52	25	2477.0973	1274.68118	12
R	53	26	2633.19841	1173.6335	11
N	54	27	2747.24133	1017.53239	10
A	55	28	2818.27845	903.48946	9
N	56	29	2932.32137	832.45235	8
A	57	30	3003.35849	718.40942	7
N	58	31	3117.40141	647.37231	6
L	59	32	3230.48548	533.32938	5
A	60	33	3301.52259	420.24532	4
T	61	34	3402.57027	349.20821	3
T	62	35	3503.61795	248.16053	2
K	63	36	3631.71291	147.11285	1

Table S6 - Theoretical mass fragments of peptide C2$_{28\text{-}63}$G^{61} [M+H]$^{+}$

			b	*y*	
**A**	28	1	72.04444	3605.69726	36
**V**	29	2	171.11285	3534.66015	35
**V**	30	3	270.18126	3435.59173	34
**E**	31	4	399.22386	3336.52332	33
**N**	32	5	513.26678	3207.48073	32
**S**	33	6	600.29881	3093.4378	31
**H**	34	7	737.35772	3006.40577	30
**N**	35	8	851.40065	2869.34686	29
**A**	36	9	922.43776	2755.30393	28
**G**	37	10	979.45923	2684.26682	27
**P**	38	11	1076.51199	2627.24536	26
**A**	39	12	1147.5491	2530.19259	25
**Y**	40	13	1310.61243	2459.15548	24
**C**	41	14	1413.62162	2296.09215	23
**V**	42	15	1512.69003	2193.08296	22
**G**	43	16	1569.71149	2094.01455	21
**Y**	44	17	1732.77482	2036.99309	20
**C**	45	18	1835.78401	1873.92976	19
**G**	46	19	1892.80547	1770.92057	18
**N**	47	20	2006.8484	1713.89911	17
**N**	48	21	2120.89133	1599.85618	16
**G**	49	22	2177.91279	1485.81326	15
**V**	50	23	2276.9812	1428.79179	14
**V**	51	24	2376.04962	1329.72338	13
**T**	52	25	2477.0973	1230.65497	12
**R**	53	26	2633.19841	1129.60729	11
**N**	54	27	2747.24133	973.50618	10
**A**	55	28	2818.27845	859.46325	9
**N**	56	29	2932.32137	788.42614	8
**A**	57	30	3003.35849	674.38321	7
**N**	58	31	3117.40141	603.34609	6
**L**	59	32	3230.48548	489.30317	5
**A**	60	33	3301.52259	376.2191	4
**G**	61	34	3358.54406	305.18199	3
**T**	62	35	3459.59173	248.16053	2
**K**	63	36	3587.6867	147.11285	1

Table S7 - Theoretical mass fragments of peptide C2$_{28-63}$A^{61}*d4*

		b	*y*	
**A**	1	72.04444	3623.73802	36
**V**	2	171.11285	3552.70091	35
**V**	3	270.18126	3453.63249	34
**E**	4	399.22386	3354.56408	33
**N**	5	513.26678	3225.52149	32
**S**	6	600.29881	3111.47856	31
**H**	7	737.35772	3024.44653	30
**N**	8	851.40065	2887.38762	29
**A**	9	922.43776	2773.34469	28
**G**	10	979.45923	2702.30758	27
**P**	11	1076.51199	2645.28612	26
**A**	12	1147.5491	2548.23335	25
**Y**	13	1310.61243	2477.19624	24
**C**	14	1413.62162	2314.13291	23
**V**	15	1512.69003	2211.12372	22
**G**	16	1569.71149	2112.05531	21
**Y**	17	1732.77482	2055.03385	20
**C**	18	1835.78401	1891.97052	19
**G**	19	1892.80547	1788.96133	18
**N**	20	2006.8484	1731.93987	17
**N**	21	2120.89133	1617.89694	16
**G**	22	2177.91279	1503.85402	15
**V**	23	2276.9812	1446.83255	14
**V**	24	2376.04962	1347.76414	13
**T**	25	2477.0973	1248.69573	12
**R**	26	2633.19841	1147.64805	11
**N**	27	2747.24133	991.54694	10
**A**	28	2818.27845	877.50401	9
**N**	29	2932.32137	806.4669	8
**A**	30	3003.35849	692.42397	7
**N**	31	3117.40141	621.38685	6
**L**	32	3230.48548	507.34393	5
**A**	33	3301.52259	394.25986	4
**A**	34	3376.58482	323.22275	3
**T**	35	3477.63249	248.16053	2
**K**	36	3605.72746	147.11285	1

Table S8 - Theoretical Mass fragments of peptide C2$_{28\text{-}63}$A^{61}*d3*

		b	*y*	
**A**	1	72.04444	3622.73174	36
**V**	2	171.11285	3551.69463	35
**V**	3	270.18126	3452.62621	34
**E**	4	399.22386	3353.5578	33
**N**	5	513.26678	3224.51521	32
**S**	6	600.29881	3110.47228	31
**H**	7	737.35772	3023.44025	30
**N**	8	851.40065	2886.38134	29
**A**	9	922.43776	2772.33841	28
**G**	10	979.45923	2701.3013	27
**P**	11	1076.51199	2644.27984	26
**A**	12	1147.5491	2547.22707	25
**Y**	13	1310.61243	2476.18996	24
**C**	14	1413.62162	2313.12663	23
**V**	15	1512.69003	2210.11744	22
**G**	16	1569.71149	2111.04903	21
**Y**	17	1732.77482	2054.02757	20
**C**	18	1835.78401	1890.96424	19
**G**	19	1892.80547	1787.95505	18
**N**	20	2006.8484	1730.93359	17
**N**	21	2120.89133	1616.89066	16
**G**	22	2177.91279	1502.84774	15
**V**	23	2276.9812	1445.82627	14
**V**	24	2376.04962	1346.75786	13
**T**	25	2477.0973	1247.68945	12
**R**	26	2633.19841	1146.64177	11
**N**	27	2747.24133	990.54066	10
**A**	28	2818.27845	876.49773	9
**N**	29	2932.32137	805.46062	8
**A**	30	3003.35849	691.41769	7
**N**	31	3117.40141	620.38057	6
**L**	32	3230.48548	506.33765	5
**A**	33	3301.52259	393.25358	4
**A**	34	3375.57854	322.21647	3
**T**	35	3476.62621	248.16053	2
**K**	36	3604.72118	147.11285	1

Supplementary references

1. Waterhouse, A. M., Procter, J. B., Martin, D. M., Clamp, M., and Barton, G. J. (2009) Jalview Version 2--a multiple sequence alignment editor and analysis workbench. *Bioinformatics*. **25**, 1189-1191
